# Automated Protein Secondary Structure Assignment from C*α* Positions Using Neural Networks

**DOI:** 10.3390/biom12060841

**Published:** 2022-06-17

**Authors:** Mohammad N. Saqib, Justyna D. Kryś, Dominik Gront

**Affiliations:** Faculty of Chemistry, Biological and Chemical Research Center, University of Warsaw, Pasteura 1, 02-093 Warsaw, Poland; m.saqib@student.uw.edu.pl (M.N.S.); jkrys@chem.uw.edu.pl (J.D.K.)

**Keywords:** deep learning, machine learning, multi-class classifier, neural networks, protein secondary structure, protein structure prediction, protein secondary structure assignment

## Abstract

The assignment of secondary structure elements in protein conformations is necessary to interpret a protein model that has been established by computational methods. The process essentially involves labeling the amino acid residues with H (Helix), E (Strand), or C (Coil, also known as Loop). When particular atoms are absent from an input protein structure, the procedure becomes more complicated, especially when only the alpha carbon locations are known. Various techniques have been tested and applied to this problem during the last forty years. The application of machine learning techniques is the most recent trend. This contribution presents the HECA classifier, which uses neural networks to assign protein secondary structure types. The technique exclusively employs Cα coordinates. The Keras (TensorFlow) library was used to implement and train the neural network model. The BioShell toolkit was used to calculate the neural network input features from raw coordinates. The study’s findings show that neural network-based methods may be successfully used to take on structure assignment challenges when only Cα trace is available. Thanks to the careful selection of input features, our approach’s accuracy (above 97%) exceeded that of the existing methods.

## 1. Introduction

In the 1950s, Pauling and Corey identified the presence of regular substructures in proteins called α-helices (H) and β-sheets (E) [1], which are connected with loops (C). At first, these regions were manually assigned based on a visual inspection of the protein’s main chain geometry. Over the years, several techniques have been devised to automate the assignment process. The DSSP [2] algorithm, devised in the 1980s, achieves its objective by detecting hydrogen bonds along the protein chains. This method expanded the categorization from three to eight classes: $3_{10}$ helices (G), α-helices (H), π-helices (I), β-strands (E), β-bridges (B), turns (T), bends (S), and others (C), where some states, such as I, G, and B, are relatively infrequent. However, residue coverage for DSSP assignments is poor when the structure is not well-defined or not well-ordered, and the stringent criteria set for hydrogen bonds are not met. STRIDE [3] used a modified hydrogen-bond energy function and included backbone dihedral angles in its algorithm to address this issue. These two methods are typically seen as the reference definition for secondary structure assignment. Indeed, the all-atom information allows a very accurate description of the hydrogen bonding patterns between C=O and N-H groups of the protein backbone and leads to the assignment of Secondary Structure Elements (SSEs) in the spirit of Pauling and Corey’s observations.

Unfortunately, on many occasions, all-atom representation is not available. A number of methods, therefore, have been devised to assign a secondary structure solely from Cα coordinates, e.g., DEFINE_S [4], P-CURVE [5], PROSIGN [6], SACF [7], P-SEA [8], PALSSE [9], STICK [10], VoTAP [11], SABA [12] and SST [13]. These algorithms can be grouped into three categories:

(i) Methods that assign SSE directly from local geometric parameters derived from Cα positions, e.g., local distances along the chain (DEFINE_S, SABA, STICK), possibly combined with information about close spatial neighbors (P-SEA), dihedral angles (PALSSE), and contact map (VoTAP); fitting a curve to Cα points was also utilized [6,7].
(ii) Methods that cut the query structure into short Cα structural fragments and compare them to structural fragments extracted from known protein structures, e.g., by means of trained Bayesian (SST) or a Nearest-Neighbor Classifier (SACF).
(iii) Methods that use Machine Learning (ML) to infer SSE. The progress in the field of ML observed in the twenty-first century significantly increased the popularity of these methods in bioinformatics in general, including the problem of the secondary structure assignment. The PCASSO [14] method uses the Random Forest classifier technique, where 16 features are solely based on Cα coordinates. It reports a very high accuracy reaching 96% with respect to DSSP. Another Random Forest-based approach RaFoSA [15] uses 30 features: 1 × residue type, 6 × Cα-Cα distances, the angle between three Cα atoms, 4 × torsional angles formed by four Cα atoms, and the number of Cα-Cα contacts. Similarly, it is also 96% accurate with respect to DSSP. More sophisticated methods employ Neural Networks [16] and Convolutional Neural Networks (CNN), as observed in the DLFSA [17]. The accuracy reported by the authors in the latter case is somewhat lower: around 83% depending on the PDB files. A consensus approach was also described, where the final assignment was decided as a consensus of four different ML techniques [18].

Unfortunately, many of these methods are no longer being maintained. At present, SABA is only limited to a web server, which allows users to analyze only one PDB file at a time. Furthermore, many of the approaches (e.g., DEFINE_S, DLFSA, and P-CURVE) feature relatively low accuracy: below 90%. Other methods that are still actively maintained are PALSSE, SST, PSSPRED, PROSS, and PCASSO. At the same time, coarse-grained methods are still widely applied in protein modeling. Since the seminal work of Levitt and Warshel [19], the modeling and dynamics of the coarse-grained structures have been rapidly growing and inspiring biomolecular modeling for a few decades now. The very recent introduction of ML approaches such as AlfaFold2 [20] and RosettaFold [21] has colossally influenced and dramatically changed the field of protein structure prediction. However, to date, multiscale algorithms remain widely used methods to study long-time protein dynamics and aggregation [22]. The original Levitt–Warshel model has been continuously used, and its refined version has been recently published [23]. Another very successful and popular approach is the Martini force field. Initially proposed for lipid systems, it has been extended to proteins [24] and has been used worldwide to simulate a variety of biological systems [25]. A detailed description of the current progress in coarse-grained and multiscale modeling methods can be found in a very recent review article [26].

Applications of CG methods primarily focus on modeling the dynamics of large and/or partially unstructured systems such as intrinsically disordered proteins [27,28], prions, protein–peptide binding [29] and viral capsids [30]. In particular, the applications of CG models also include modeling of large bio-macromolecular complexes based on low-resolution experimental data, such as cry-EM maps [31]. Many of the CG methods rely on Cα positions to define reduced protein representations, e.g., CABS [32], UNRES [26] or AWSEM [27,33]. Typically, secondary structure assignment is one of the first steps to analyze trajectories produced by such tools, followed by reconstruction of the all-atom representation. For instance, both PCASSO and P-SEA techniques were used for protein simulations—the first method used CG to analyze protein interactions with nanoparticles [34], and the latter processed α-synuclein aggregation [35].

The newest research directions focus on the generalization of CG force fields and their parametrization for biomacromolecular systems. These systems are generally comprised of proteins, nucleic acids, and polysaccharides, e.g., MARTINI and UNICORN [36]. The success of ML methods in the field mentioned above helped close a long-lasting chapter of protein structure prediction. However, it opened new avenues, such as training a deep machine model to learn CG potentials. Therefore, this progress in multiscale modeling methods increases the demand for accurate and reliable methods that assign secondary structure to protein conformations in a reduced representation and for fast and accurate reconstruction of atomic details from Cα coordinates. Therefore, efforts were made to develop a new, open-source solution to this problem that will be easily accessible.

## 2. Methods

In this contribution, an approach called HECA (H-E-C Assigner) is proposed to assign protein secondary structures only from Cα trace through the application of the artificial neural network.

### 2.1. Network Architecture

**Figure 1:**
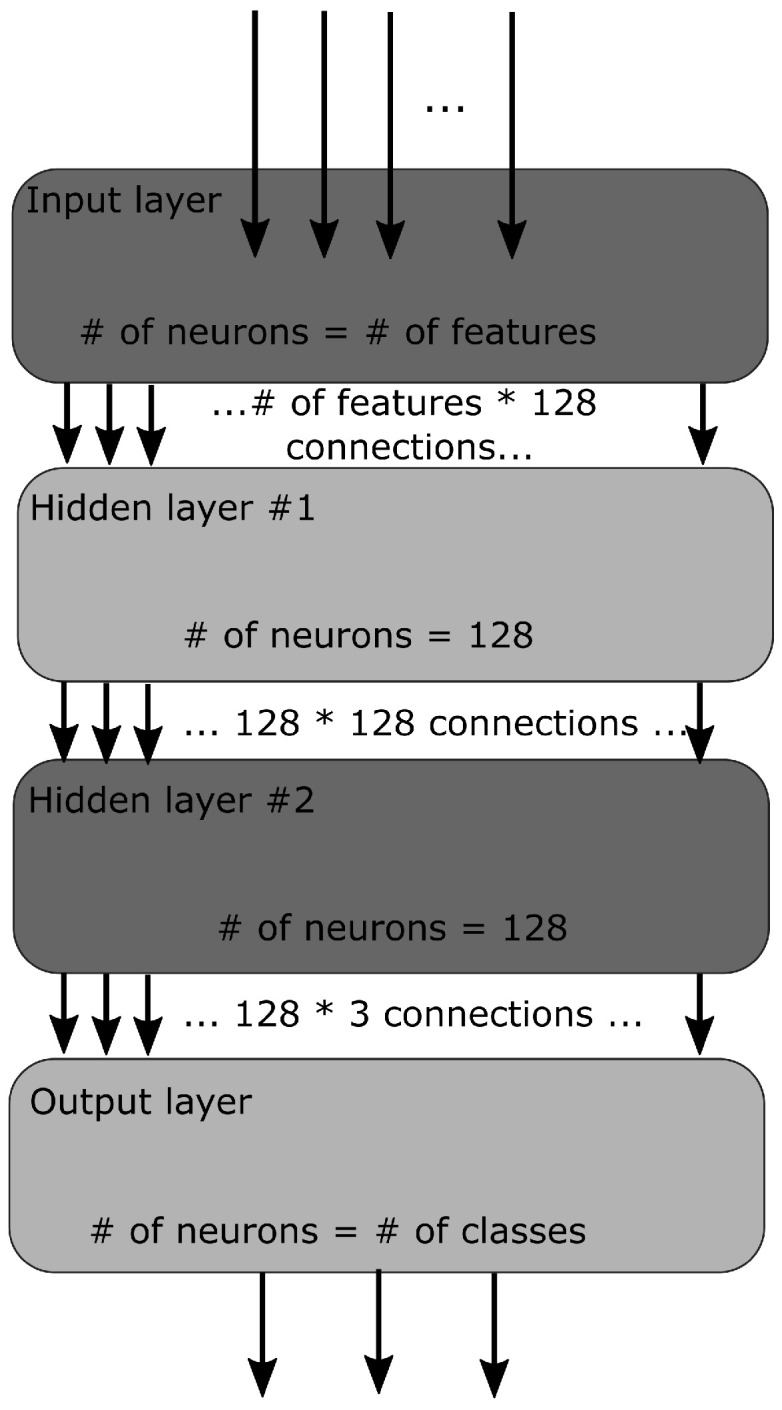
The network architecture. The neural network consists of an input, two hidden, and an output layer.

A simple MLP (Multi-Layered Perceptron) has been used in this work. An MLP is a type of neural network where the connections between layers are only feed-forward. The experiment was started with four layers, where there was one input layer, one output layer, and two hidden layers. The architecture of our network is graphically presented in Figure 1. The input layer takes features (see below for details) computed over N Cα atoms corresponding to a contiguous N-residue segment, i.e., N-peptide structure. The output layer had three neurons corresponding to the three classes H, E, and C to be predicted for the middle residue of a segment. The following values of N were tested in our study: 5, 7, 9, 11, and 13; for practical reasons, only odd values of N were used. As shown by Kůrková [37], two hidden layers should be used to make up for the loss of efficiency when regular activation functions are used. Therefore, in this study, a second hidden layer aims to reduce the total number of hidden nodes substantially. Initially, the MLP had 32 × 32 hidden neurons, a learning rate of 0.001, a batch size of 10, and an epoch size of 1000. Then gradually, 64 × 64, 96 × 96 neurons were tested, and finally, the experiment settled on 128 × 128 neurons with a learning rate of 0.01 and 3500 epochs as they gave the best results. In total, 80% of data was used for training, while the remaining 20% was for testing. When the training was complete, separate data sets were generated for validation.

Sigmoid functions (also known as logistic functions) were used to activate input and hidden layers, while the softmax function was used in the output layer of the network model. The softmax function converts a vector of K real values into another vector of K real values that results in 1 when added together. The softmax function is meant for the neural networks that predict multinomial probability distributions. In other words, the softmax function is used in multi-class classification problems involving more than two classes. The output layer of our network contains three neurons to classify the middle residue of the N-residue segment into either of the three classes. The SDG optimizer was used to optimize the weights of the network. The categorical cross-entropy loss function was used to assess the prediction error during training, which is the standard approach when the classes are one-hot-encoded.

### 2.2. Software Implementation

The training calculations were performed with Keras and TensorFlow [38] libraries accessed from a Python script. The final HECA tool (i.e., the actual predictor) was implemented in the BioShell package for structural bioinformatics written in C++, which has been continuously developed and maintained in our laboratory since 2006. Later on, TensorFlow was replaced with the frugally-deep library to minimize the size of the final executable HECA. The HECA method was also published as a web server to make it easily accessible to users. PDB-formatted text input should be provided by a user, for which the server returns a predicted secondary structure as a single string in a 3-letter code. BioShell has included Python bindings since its last version, 3.0 [39]. Therefore, the Flask framework was used on the server-side to call the appropriate Python functions bound to the C++ library. The client part was also implemented in Python, which runs in a web browser with the help of the Brython translator (https://brython.info, accessed on 17 June 2022). Finally, the VisuaLife library [40] was used to render the prediction results on the web browser.

### 2.3. Data Sets

Currently, the PDB website lists about 190,000 available deposits. However, these protein structures are redundant to a large extent. Therefore, for training, a PISCES subset [41] with protein chains identical in no more than 40% was used; the set was also restricted to deposits of resolution 1.6 Å or better and R-factor lower than 0.25. These criteria yielded a set of 6695 chains; the complete list is available from the PISCES website. The BioShell package [42,43] was used to calculate input features from Cα atoms of these chains, as described in the following subsection. Structural analysis of PDB deposits is the main application of the BioShell software, which has been developed for over a decade. The package provides numerous filters to detect incorrect or incomplete fragments of a protein chain, such as missing residues, i.e., chain breaks, missing any backbone atom, or severe stereochemical errors. Each N-residue fragment has been screened using these filters and removed if any of them failed. These filters reduced the training set to 6396 chains; the full list is available in supporting materials. The test set has been compiled by selecting one remote homolog for each chain from the training set. The search has been conducted with Jackhammer tool against the set of the sequences available from PDB deposits. For each query, we randomly selected one sequence with an e-value around $10^{-7}$. This pool of chains set was subsequently filtered to remove close homologs and incorrect entries, as described above for the training set. As a result, we compiled a test set of 4401 entries. Lists of proteins included both in the training and the test set are provided in the Supporting Materials section.

### 2.4. Input Features

**Figure 2:**
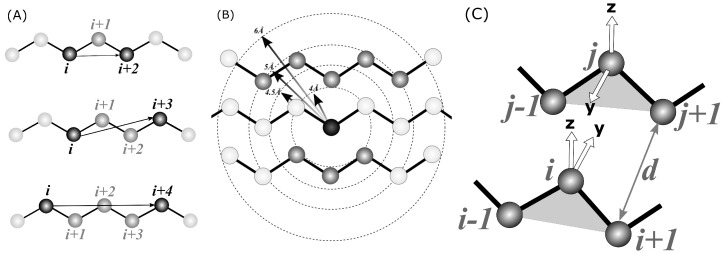
The spatial features computed from Cα positions that are used in the HECA method: (**A**) local distances between *i*-th and (*i* + 2), (*i* + 3) and (*i* + 4) atoms, (**B**) the number of spatial neighbors and (**C**) the number of hydrogen bonds in the Cα-only definition.

Before applying any ML method, a set meaningful to the nature of a problem, i.e., rotational-invariant features, must be computed from the raw coordinates of Cα atoms. Three types of features were employed in the study:

(i) Local $r_{i,i+2}$, $r_{i,i+3}^{*}$, and $r_{i,i+4}$, distances measured between Cα atoms along a contiguous segment, referred to as **local** in the text (see Figure 2A). The $r_{i,i+3}^{*}$ is a signed value, i.e., it is the distance between atoms; *i* and i+3 are multiplied by the sign of $\left(\vec{v_{i}}\times \vec{v_{i+1}}\right)\vec{v_{i+3}}$. This allows the classifier to distinguish between left-handed and right-handed conformations. All possible such distances were calculated within a segment, e.g., for N=7 five $r_{i,i+2}$, four $r_{i,i+3}^{*}$, and three $r_{i,i+4}$ were included in the input tensor.
(ii) The number of spatial neighbors found around each Cα atom of a segment within a given distance, referred to as **neighbors** in the text (see Figure 2B). Four distance cutoffs were used: 4, 4.5, 5, and 6 Å. Therefore, for N=7, the input tensor includes 7×4=28 contact counts. For example, in Figure 2B, the middle Cα atom (darkest gray) has eight neighbors within a 5 Å radius (medium-dark). Atoms separated by at most two residues along the sequence are also not included in the count (light gray).
(iii) The number of hydrogen bonds formed by each Cα atom of a segment, referred to as **hbonds** in the text. A coarse-grained hydrogen-bonding model, which has recently been developed in our laboratory (*submitted*) for the SURPASS algorithm [44,45], was used for detecting such bonds (see Figure 2C). Detailed derivation and assessment of the potential will be published elsewhere; a summary of the algorithm is provided in the Supporting Materials. In brief, a triangle is constructed from every three subsequent Cα atoms. According to our coarse-grained definition, a given triangle may form a hydrogen bond with another triangle when specific geometry criteria are met, i.e., when the two triangles are roughly parallel. Therefore, the criteria for such a hydrogen-bonding event are based on the mutual orientation of two local coordinate systems constructed on the two Cα triangles. Respective geometric criteria were derived from PDB statistics so that they match the all-atom hydrogen bonds observed in the PDB deposits. According to our model, each residue located in a beta-strand may form up to two such hydrogen bonds on either side of the triangle. Therefore, the HECA input tensor for a fragment of N residues contains N integer hydrogen bond counts that are either 0, 1, or 2.

**Figure 3:**
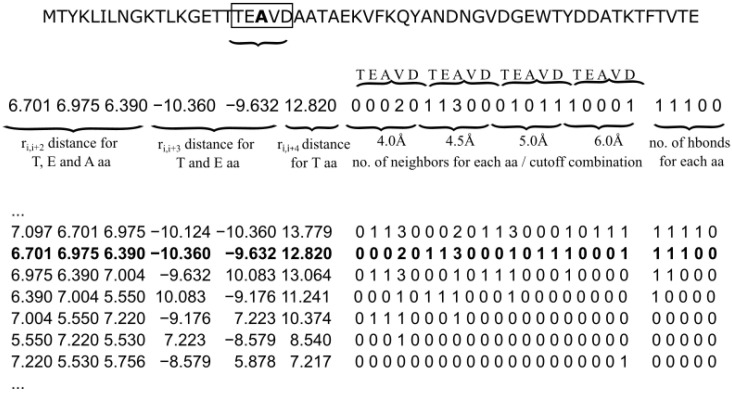
The overview of the HECA prediction. A vector of input values is computed from a given Cα-only structure for each N-residue fragment (here N=5). Each input row is used to assign H, E, and C classes to the middle residue of a segment. For example, the input row marked in bold font corresponds to a segment of TEAVD residues of 2gb1 deposit and predicts the secondary structure for the middle alanine.

The general motivation for choosing features was to provide an ML model with meaningful values that segregate the three H, E, and C classes. Structure of the input data as well as its alignment is shown in Figure 3.

## 3. Results and Discussion

Three different sets of input features were used to train the neural network model. Table 1 summarizes the training outcomes, providing Q3 averaged accuracy for each of these cases.

As expected, the more features are used, the better the prediction. Similarly, longer fragments yield better accuracy. However, the improvement made by moving from 11-mers to 13-mers is marginal. Interestingly, **local** distances are already a pretty good discriminator between the secondary structure types. Even for the shortest fragments N=5 and only **local** features, the method reaches 83.6% accuracy, which means that the Cα geometry of a helical pentapeptide differs considerably from its extended counterpart. The label H is the easiest to predict due to its unique, tight geometry that can be easily described even by local distances alone. However, the distinction between an extended coil fragment and a beta-strand is more subtle. Extended polypeptide chain fragments can often be found in loops, and their local geometry might be very similar to twisted strands. Therefore, the proper assignment of secondary structure would require detecting a regular pattern of hydrogen bonds. This information, however, is not available in Cα-only representation. The majority of methods applied so far to this problem used statistics describing close-range neighbors, i.e., counts how many other Cα can be found within a certain cutoff distance to the Cα under consideration. This is motivated by the observation that less-tightly packed fragments have a higher chance of being a loop, which typically occurs in the outer parts of a protein where it forms fewer interatomic contacts. To the contrary, regular secondary structure elements are often found in a densely packed protein core and have more spatial neighbors. In this work, the number of Cα neighbors within a 4, 4.5, 5, and 6 Å radius from a given Cα were counted. These features raise the fraction of successfully assigned H, E, and C classes by more than 5% points for the shortest segments (N=5) and by approximately 2% points for N=11 and N=13.

**Figure 4:**
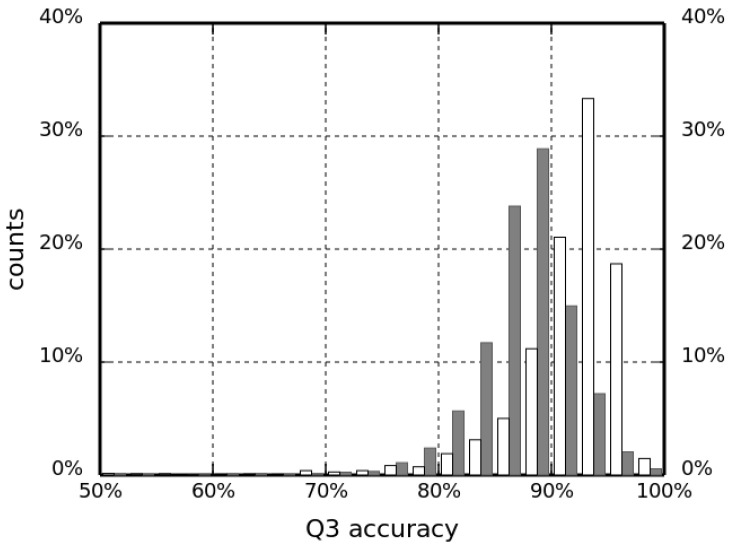
Q3 accuracy of the HECA method compared to the PCASSO approach (white and dark bars, respectively).

This improvement in the classification accuracy shows that, indeed, the information about non-local interactions can differentiate between the different SSEs. This information, however, does not encode the specific geometry of interacting protein chain segments. For example, the hydrogen-bonding pattern imposes a specific spatial arrangement of atoms that cannot be encoded by only counting the closest Cα neighbors in space. Therefore, a coarse-grained description of a hydrogen bond based on Cα coordinates was also included to improve the classification further. Even though positions of backbone atoms responsible for hydrogen bonding are not available, the arrangement of Cα atoms in the Cartesian space is tightly restricted by the geometry of SSEs. One can thus consider the count of such coarse-grained hydrogen bonds as the count of Cα atom neighbors, restricted to the very specific locations, that are relevant to a given secondary structure type (H or E). This spatial dependency makes our H-bond features improve the overall predicted accuracy (as measured by Q3), although the increase is not very high. Indeed, including this information increased the success rate in validation runs by another 2.6 and 2.8 percent points for fragment lengths N=11 and N=13, respectively. A summary assessment of the HECA method for selected values of fragment length *N* are given in Table 2. The success rate computed for the test set (4401 chains) for the best predictor is 97.48%, which is one of the highest values ever reported in the literature to date. Moreover, in Figure 4, we present a histogram of Q3 accuracy obtained on the test set by HECA and PCASSO methods (white and dark bars, respectively) with bins 2.5 percent points wide. The distribution of HECA results are shifted towards the larger Q3 values; the most populated bin is 92.5–95.0% with 1463 observations (87.5–90.0% with 1268 observations, respectively, for PCASSO).

Confusion matrices (Table 3) show that misprediction of true E as C is more common than predicting E when C is the correct answer. Information on spatial neighbors and hydrogen bonds (**neighbors** and **hbonds** features) helps resolve these ambiguities. Including this additional information improves the classification of E and C types. For example, the confusion matrix for the local-only variant of the predictor at N=13, for 13.63% of strands (E) returns C. Including the non-local features lowers this misprediction rate to 3.49% (also for N=13).

An interesting comparison can be made with the work of Sallal et al. [18]. This group has trained a very sophisticated classifier, essentially based on a logistic regression of four distinct ML approaches: Random Forest, Support Vector Machine, Multilayer Perceptron, and eXtreme Gradient Boosting. Any of these four classifiers alone is probably capable of solving the SSE assignment problem. Their approach, however, is solely based on local features such as distances and angles; they achieved 93% accuracy as to the PDB annotation, which is very similar to the classification by a simpler network described in this contribution. In another very recent work, Nasr et al. [16] described a deep neural network that is quite similar to the HECA method. Their approach employed a network that is much deeper and wider (6 hidden layers, up to 379 neurons) than that of HECA (2 hidden layers, 128 neurons each). Nasr et al. utilized both local features (angles and distances) as well as the number of spatial neighbors and achieved Q3=0.931 on their set of 72 chains, while HECA reached Q3=0.942 on the very same benchmark set (see Table 4 for detailed comparison). These two examples underpin the importance of non-local features, most importantly, the coarse-grained hydrogen-bond model utilized in this work.

## 4. Summary

Despite the recent progress in atomistic simulations of biological molecules, coarse-grained modeling is still an invaluable method in computational biophysics [46]. They facilitate studies at a biologically meaningful timescale with limited available computation power. Algorithms for secondary structure assignment from Cα-only representation, such as the HECA method presented in this contribution, are essential tools for analyzing the results of these simulations. Moreover, coarse-grained methods are often combined with higher accuracy approaches into multiscale modeling protocols [47]. Such an approach was also employed to fit protein chains into EM electron density when solving challenging targets [31]. Proper secondary structure annotation is very helpful to reconstruct all-atom representation from the reduced one [48] and may have a significant impact on the whole multiscale protocol.

The HECA method could also be helpful in detecting secondary structure elements in experimental structures. At present, about 0.5% of all protein chains (or 0.6% of all residues) available from the PDB contain only Cα atoms. This problem is indeed not very common. The majority of these deposits are quite old, as the crystallography methods have significantly improved during the past decades and can nowadays deliver structures of sub-angstrom accuracy. However, for challenging targets such as fibers (1EI3, 2000), collagen (3HQV, 2009), spliceosomes (5O9Z, 2017), ribosomes (4ADX, 2012) or viruses (7V3I, 2021), experimental methods such as electron microscopy can provide only a low-resolution structural description. Overall, we found nineteen Cα-only deposits added to PDB since January 2020, all of them established by EM. Delineation of a Cα into secondary structure segments is often the first step in fast protein structure comparison and alignment procedures. This may also be the case for computed models. By providing both a stand-alone package published under the Apache 2.0 Open Source license and a web server, we believe the method will be easy to apply and widely accepted in the field.

## Figures and Tables

**Table 1:** The Q3 accuracy on the training and validation set for the HECA neural network with different input data sets (in percentages).

Fragment Length	Local		Local + Neighbors		Local + Neighbors + Hbonds	
	Training	Validation	Training	Validation	Training	Validation
5	83.58	83.60	88.93	88.75	91.91	91.98
7	89.89	89.99	96.20	96.39	95.40	95.48
9	91.84	91.88	94.03	94.10	96.85	96.91
11	92.37	92.46	94.51	94.57	97.29	97.33
13	92.53	92.61	94.68	94.89	97.39	97.48

**Table 2:** The summary of the HECA algorithm performance measured on a test data set.

Fragment length	5	7	9	11	13
No. of ideally predicted proteins	144	246	406	465	491
Differences between predicted and true classes	H: 6.97%	H: 2.87%	H: 2.78%	H: 2.05%	H: 1.88%
	E: 5.16%	E: 4.55%	E: 4.30%	E: 3.44%	E: 3.63%
	C: 2.27%	C: 7.69%	C: 7.69%	C: 4.50%	C: 4.44%
Average differences	8.13%	5.03%	3.70%	3.33%	3.31%

**Table 3:** Confusion matrices for all (**local** + **neighbors** + **hbonds**) features and segments N=5,11,13 compared to **local** features and N=13 (bottom right).

5-mer	Predicted			11-mer	Predicted		
	H	E	C		H	E	C
H	93.026%	0.035%	6.938%	H	97.953%	0.036%	2.009%
E	0.180%	94.840%	4.978%	E	0.123%	96.559%	3.317%
C	11.089%	1.179%	87.731%	C	2.771%	1.730%	95.498%
**13-mer**	**Predicted**			**13-mer**	**Predicted**		
	**H**	**E**	**C**		**H**	**E**	**C**
H	98.11%	0.028%	1.859%	H	97.680%	0.205%	2.114%
E	0.134%	96.37%	3.49%	E	0.684%	85.680%	13.634%
C	2.80%	1.634%	95.563%	C	3.398%	7.043%	89.557%

**Table 4:** Q3 accuracy of the HECA method compared with PCASSO and results by Nasr et al. [16].

PDB	Nres	Type	HECA	PCASSO	Nasr et al.	PDB	Nres	Type	HECA	PCASSO	Nasr et al.
1EAR_A	135	β	0.957	0.936	0.956	4MNC_A	299	α+β	0.950	0.849	0.930
1GQI_A	702	α	0.971	0.879	0.925	4MYD_A	246	α+β	0.972	0.857	0.947
1NUY_A	322	α+β	0.960	0.859	0.891	4OH7_A	296	α	0.953	0.427	0.909
1OK0_A	68	β	0.972	0.972	0.926	4P3H_A	184	α+β	0.921	0.473	0.918
1SDI_A	207	α	0.962	0.901	0.981	4WKA_A	363	α+β	0.962	0.853	0.909
1UJ8_A	66	α	0.917	0.794	0.939	4ZDS_A	125	α	0.939	0.825	0.960
1Z6N_A	160	α	0.945	0.867	0.950	5CKL_A	175	α	0.933	0.883	0.937
2FGQ_X	324	β	0.954	0.921	0.920	5CL8_A	225	α	0.948	0.852	0.951
2FP1_A	159	α	0.963	0.890	0.981	5CVW_A	142	β	0.946	0.773	0.831
2FVY_A	298	α+β	0.977	0.898	0.953	5GZK_A	412	α	0.935	0.861	0.876
2I5V_O	239	β	0.979	0.951	0.954	5JUH_A	130	α+β	0.948	0.627	0.862
2JDA_A	131	β	0.920	0.791	0.756	5LT5_A	198	α+β	0.960	0.877	0.909
2O1T_A	428	α+β	0.907	0.847	0.930	5T9Y_A	312	α+β	0.915	0.598	0.933
2OPC_A	109	β	0.956	0.930	0.908	5TIF_A	176	α	0.961	0.839	0.955
2QKV_A	85	α+β	0.923	0.945	0.976	5UEB_A	136	α+β	0.943	0.829	0.956
2RIN_A	282	α+β	0.895	0.788	0.901	5W53_A	297	α	0.963	0.947	0.970
2RIQ_A	129	α	0.888	0.903	0.938	5WEC_A	104	β	0.946	0.866	0.981
2Z6R_A	256	α+β	0.958	0.852	0.930	5YDE_A	105	α+β	0.936	0.891	0.924
2ZDP_A	104	α+β	0.972	0.798	0.904	5ZIM_A	222	α	0.942	0.912	0.959
3BQP_A	74	α	0.937	0.900	1.000	6A2W_A	159	α	0.957	0.831	0.975
3D2Y_A	251	α+β	0.968	0.914	0.952	6E7E_A	163	α	0.970	0.905	0.982
3DXY_A	200	α+β	0.932	0.869	0.970	6ER6_A	82	α	0.931	0.886	0.976
3KYJ_A	123	α	0.961	0.658	0.992	6GEH_A	250	α+β	0.968	0.910	0.956
3LFK_A	115	α+β	0.909	0.811	0.930	6I1A_A	352	α	0.946	0.731	0.932
3NJN_A	108	β	0.903	0.517	0.861	6IY4_I	86	α+β	0.881	0.838	0.907
3OBQ_A	135	α+β	0.950	0.907	0.956	6JH9_B	22	α	0.655	0.689	0.773
3Q40_A	169	α	0.964	0.946	0.975	6JM5_A	114	α+β	0.918	0.886	0.904
3R87_A	125	α+β	0.984	0.916	0.960	6JU1_A	387	α+β	0.961	0.785	0.925
3RT2_A	165	α+β	0.959	0.935	0.964	6JWF_A	400	β	0.973	0.899	0.915
3V4K_A	180	α+β	0.967	0.672	0.967	6KTK_A	362	α+β	0.956	0.495	0.945
3VK5_A	247	α+β	0.956	0.889	0.976	6NEY_A	119	α	0.936	0.880	0.933
3VMK_A	363	α+β	0.924	0.523	0.898	6NZS_A	581	β	0.950	0.873	0.880
3WDN_A	119	α+β	0.952	0.872	0.933	6P80_A	312	α	0.952	0.874	0.942
4AYO_A	428	α	0.965	0.875	0.914	6TM6_A	90	β	0.855	0.876	0.878
4B20_A	264	α+β	0.950	0.662	0.930	6TZX_A	217	α	0.950	0.865	0.926
4GMU_A	604	α+β	0.960	0.863	0.904	6ULO_A	310	α	0.940	0.340	0.923
4JUI_A	463	α+β	0.970	0.704	0.935	6YDR_A	122	α	0.976	0.860	0.992
4L9E_A	108	α+β	0.947	0.904	0.917	**average**			0.942	0.820	0.931

## Data Availability

Not applicable.

## Supplementary Materials

The following are available at https://www.mdpi.com/article/10.3390/biom12060841/s1, Table SUP1: Test set, Table SUP2: Train set, SUP3: Hbonds definition for HECA.

## Sample Availability

Standalone distribution of the HECA program is available as a part of the BioShell package, which source code is available from the git repository: https://bitbucket.org/dgront/bioshell, 17 June 2022. The HECA server is available on the following address: https://bioshell.pl/software-servers/heca, 17 June 2022.

## References

[1] Pauling L., Corey R.B., Branson H.R. (1951). The structure of proteins: Two hydrogen-bonded helical configurations of the polypeptide chain. Proc. Natl. Acad. Sci. USA.

[2] Kabsch W., Sander C. (1983). Dictionary of protein secondary structure: Pattern recognition of hydrogen-bonded and geometrical features. Biopolymers.

[3] Frishman D., Argos P. (1995). Knowledge-based protein secondary structure assignment. Proteins.

[4] Richards F.M., Kundrot C.E. (1988). Identification of structural motifs from protein coordinate data: Secondary structure and first-level supersecondary structure. Proteins Struct. Funct. Bioinform..

[5] Sklenar H., Etchebest C., Lavery R. (1989). Describing protein structure: A general algorithm yielding complete helicoidal parameters and a unique overall axis. Proteins Struct. Funct. Bioinform..

[6] Hosseini S.R., Sadeghi M., Pezeshk H., Eslahchi C., Habibi M. (2008). PROSIGN: A method for protein secondary structure assignment based on three-dimensional coordinates of consecutive C*α* atoms. Comput. Biol. Chem..

[7] Cao C., Wang G., Liu A., Xu S., Wang L., Zou S. (2016). A New Secondary Structure Assignment Algorithm Using C*α* Backbone Fragments. Int. J. Mol. Sci..

[8] Labesse G., Colloc’h N., Pothier J., Mornon J.R. (1997). P-sea: A new efficient assignment of secondary structure from c*α*l trace of proteins. Bioinformatics.

[9] Majumdar I., Krishna S.S., Grishin N.V. (2005). PALSSE: A program to delineate linear secondary structural elements from protein structures. BMC Bioinform..

[10] Taylor W.R. (2001). Defining linear segments in protein structure. J. Mol. Biol..

[11] Dupuis F., Sadoc J.F., Mornon J.P. (2004). Protein Secondary Structure Assignment Through Voronoï Tessellation. Proteins Struct. Funct. Genet..

[12] Park S.Y., Yoo M.J., Shin J., Cho K.H. (2011). SABA (secondary structure assignment program based on only alpha carbons): A novel pseudo center geometrical criterion for accurate assignment of protein secondary structures. BMB Rep..

[13] Konagurthu A.S., Lesk A.M., Allison L. (2012). Minimum message length inference of secondary structure from protein coordinate data. Bioinformatics.

[14] Law S.M., Frank A.T., Brooks C.L. (2014). PCASSO: A fast and efficient C*α*-based method for accurately assigning protein secondary structure elements. J. Comput. Chem..

[15] Salawu E.O. (2016). RaFoSA: Random forests secondary structure assignment for coarse-grained and all-atom protein systems. Cogent Biol..

[16] Nasr K.A., Sekmen A., Bilgin B., Jones C., Koku A.B. Deep Learning for Assignment of Protein Secondary Structure Elements from C Coordinates. Proceedings of the 2021 IEEE International Conference on Bioinformatics and Biomedicine (BIBM).

[17] Antony J.V., Madhu P., Balakrishnan J.P., Yadav H. (2021). Assigning secondary structure in proteins using AI. J. Mol. Model..

[18] Sallal M.A., Chen W., Nasr K.A. Machine Learning Approach to Assign Protein Secondary Structure Elements from Ca Trace. Proceedings of the 2020 IEEE International Conference on Bioinformatics and Biomedicine (BIBM).

[19] Levitt M., Warshel A. (1975). Computer simulation of protein folding. Nature.

[20] Jumper J., Evans R., Pritzel A., Green T., Figurnov M., Ronneberger O., Tunyasuvunakool K., Bates R., Žídek A., Potapenko A. (2021). Highly accurate protein structure prediction with AlphaFold. Nature.

[21] Baek M., DiMaio F., Anishchenko I., Dauparas J., Ovchinnikov S., Lee G.R., Wang J., Cong Q., Kinch L.N., Dustin Schaeffer R. (2021). Accurate prediction of protein structures and interactions using a three-track neural network. Science.

[22] Sieradzan A.K., Czaplewski C., Krupa P., Mozolewska M.A., Karczyńska A.S., Lipska A.G., Lubecka E.A., Gołaś E., Wirecki T., Makowski M. (2022). Modeling the Structure, Dynamics, and Transformations of Proteins with the UNRES Force Field.

[23] Vicatos S., Rychkova A., Mukherjee S., Warshel A. (2014). An effective Coarse-grained model for biological simulations: Recent refinements and validations. Proteins Struct. Funct. Bioinform..

[24] Monticelli L., Kandasamy S.K., Periole X., Larson R.G., Tieleman D.P., Marrink S.J. (2008). The MARTINI coarse-grained force field: Extension to proteins. J. Chem. Theory Comput..

[25] Marrink S.J., Tieleman D.P. (2013). Perspective on the martini model. Chem. Soc. Rev..

[26] Liwo A., Czaplewski C., Sieradzan A.K., Lipska A.G., Samsonov S.A., Murarka R.K. (2021). Theory and Practice of Coarse-Grained Molecular Dynamics of Biologically Important Systems. Biomolecules.

[27] Wu H., Wolynes P.G., Papoian G.A. (2018). AWSEM-IDP: A Coarse-Grained Force Field for Intrinsically Disordered Proteins. J. Phys. Chem. B.

[28] Tesei G., Schulze T.K., Crehuet R., Lindorff-Larsen K. (2021). Accurate model of liquid-liquid phase behavior of intrinsically disordered proteins from optimization of single-chain properties. Proc. Natl. Acad. Sci. USA.

[29] Kurcinski M., Badaczewska-Dawid A., Kolinski M., Kolinski A., Kmiecik S. (2020). Flexible docking of peptides to proteins using CABS-dock. Protein Sci..

[30] Tan C., Jung J., Kobayashi C., Torre D.U.L., Takada S., Sugita Y. (2022). Implementation of residue-level coarsegrained models in GENESIS for large-scale molecular dynamics simulations. PLoS Comput. Biol..

[31] Kulik M., Mori T., Sugita Y. (2021). Multi-Scale Flexible Fitting of Proteins to Cryo-EM Density Maps at Medium Resolution. Front. Mol. Biosci..

[32] Kolinski A., Gront D. (2007). Comparative modeling without implicit sequence alignments. Bioinformatics.

[33] Davtyan A., Schafer N.P., Zheng W., Clementi C., Wolynes P.G., Papoian G.A. (2012). AWSEM-MD: Protein structure prediction using coarse-grained physical potentials and bioinformatically based local structure biasing. J. Phys. Chem. B.

[34] Wei S., Ahlstrom L.S., Brooks C.L. (2017). Exploring Protein–Nanoparticle Interactions with Coarse-Grained Protein Folding Models. Small.

[35] Guzzo A., Delarue P., Rojas A., Nicolaï A., Maisuradze G.G., Senet P. (2021). Missense Mutations Modify the Conformational Ensemble of the *α*-Synuclein Monomer Which Exhibits a Two-Phase Characteristic. Front. Mol. Biosci..

[36] Liwo A., Czaplewski C., Sieradzan A.K., Lubecka E.A., Lipska A.G., Golon Ł., Karczynska A., Krupa P., Mozolewska M.A., Makowski M. (2020). Scale-consistent approach to the derivation of coarse-grained force fields for simulating structure, dynamics, and thermodynamics of biopolymers. Prog. Mol. Biol. Transl. Sci..

[37] Kůrková V. (1992). Kolmogorov’s theorem and multilayer neural networks. Neural Netw..

[38] Abadi M., Agarwal A., Barham P., Brevdo E., Chen Z., Citro C., Corrado G.S., Davis A., Dean J., Devin M. (2016). TensorFlow: Large-Scale Machine Learning on Heterogeneous Distributed Systems. arXiv.

[39] Macnar J.M., Szulc N.A., Kryś J.D., Badaczewska-Dawid A.E., Gront D. (2020). BioShell 3.0: Library for Processing Structural Biology Data. Biomolecules.

[40] Kryś J.D., Gront D. (2021). VisuaLife: Library for interactive visualization in rich web applications. Bioinformatics.

[41] Wang G., Dunbrack R.L. (2003). PISCES: A protein sequence culling server. Bioinformatics.

[42] Gront D., Kolinski A. (2006). BioShell - a package of tools for structural biology computations. Bioinformatics.

[43] Gront D., Kolinski A. (2008). Utility library for structural bioinformatics. Bioinformatics.

[44] Dawid A., Gront D., Kolinski A. (2017). SURPASS Low-Resolution Coarse-Grained Protein Modeling. J. Chem. Theory Comput..

[45] Dawid A., Gront D., Kolinski A. (2018). Coarse-Grained Modeling of the Interplay between Secondary Structure Propensities and Protein Fold Assembly. J. Chem. Theory Comput..

[46] Kmiecik S., Gront D., Kolinski M., Wieteska L., Dawid A., Kolinski A. (2016). Coarse-Grained Protein Models and Their Applications. Chem. Rev..

[47] Wabik J., Kmiecik S., Gront D., Kouza M., Koliński A. (2013). Combining coarse-grained protein models with replica-exchange all-atom molecular dynamics. Int. J. Mol. Sci..

[48] Gront D., Kmiecik S., Kolinski A. (2007). Backbone building from quadrilaterals: A fast and accurate algorithm for protein backbone reconstruction from alpha carbon coordinates. J. Comput. Chem..

