# Supplementary material for: Automated Protein Secondary Structure Assignment from Cα Positions Using Neural Networks
Source: Biomolecules. 2022 Jun 17;12(6):841. doi: 10.3390/biom12060841 (PMC9220970; doi:10.3390/biom12060841)
Supplement: Supplementary file 1 [file biomolecules-12-00841-s001.zip › SUP1-test_set_4401.pdf]

1a6jA	1dgwX	1fduA	1i5gA	1kpsD	1n65A	1pvtA	1s20H
1a97A	1dhrA	1fj7A	1ia9A	1kqgC	1n7kA	1pvyA	1s3pA
1ae3A	1dinA	1fmd2	1icfD	1krtA	1n9eD	1pxzA	1s4fC
1ak0A	1dm0A	1fp0A	1idjB	1ks6A	1namA	1q0dA	1s5aA
1ak5A	1dnuD	1fp3A	1if1A	1ksiA	1narA	1q3xA	1s7jB
1akpA	1doiA	1fs2A	1ihnB	1ksqA	1nciB	1q67A	1sa8A
1am2A	1dp3A	1ft9A	1ii2B	1ku5A	1ne2A	1q7lD	1sddA
1apyA	1dq3A	1fueA	1iiuA	1kutB	1nhlA	1q8rB	1sfxB
1arbA	1dqnA	1fviA	1ijcA	1kvqA	1nlqE	1qa7A	1sh8B
1ashA	1dqxD	1g3nC	1im4A	1kvuA	1nm3B	1qcxA	1sisA
1avgI	1ds6B	1g4uS	1im8B	1kwaA	1nm6A	1qe5C	1smsA
1aw5A	1ds9A	1g71B	1ipeA	1kywF	1npyD	1qfxA	1sngA
1b5fB	1dw2B	1g7oA	1ipiB	1l0oC	1nt2A	1qgeD	1sp4A
1b7dA	1dwnA	1g8jD	1iquA	1l0qD	1ntxA	1qgeE	1spwA
1b7gO	1dxkA	1gcuA	1ir0A	1l2mA	1nviD	1qjtA	1squA
1b8gA	1dy2A	1gdeB	1irxB	1l3oA	1nvmF	1qlwB	1srvA
1b8sA	1e0bA	1gh2A	1ithB	1l3pA	1nzpA	1qmyA	1stoA
1bdjB	1e1hB	1gheB	1itpA	1l5xB	1o12B	1qozB	1suuA
1be1A	1e39A	1ghrA	1iu9A	1l6hA	1o7dB	1qqhA	1sv6E
1bh2A	1e5iA	1gjiA	1iv1A	1l7aB	1o99A	1qrjA	1svjA
1bjnB	1e5rB	1gklA	1iwmA	1l8lA	1o9hA	1qrrA	1svyA
1bjwA	1e68A	1gl4B	1iwpB	1l9dB	1o9zA	1qs0A	1sz3B
1bmxA	1earA	1gmvB	1ixlA	1leaA	1odsH	1qsdB	1szhB
1bo4A	1ebuD	1gouA	1iykA	1ljyA	1oe6B	1qstA	1sznA
1bo9A	1ee6A	1gpqA	1izcA	1lk9A	1ogyB	1qv9C	1szuA
1boeA	1ee9A	1gqpA	1j0aA	1ln0B	1oj1A	1qvrA	1t06A
1bolA	1egpA	1grjA	1j0tA	1lnsA	1ojzA	1qwxB	1t2wA
1bplA	1egpB	1gsmA	1j33A	1lq9B	1okgA	1qx8A	1t4mA
1bu3A	1ei3D	1gugA	1j3eA	1lrkA	1olpA	1qydD	1t57C
1bw5A	1ei3E	1gxlD	1j3pB	1lrzA	1omiB	1r1dB	1t62B
1byfB	1ei6D	1gxsB	1j6xA	1luaC	1oqjB	1r1gA	1t6pH
1c25A	1eifA	1gyoB	1jenB	1lw5D	1oqvC	1r30B	1t9fA
1c2nA	1eijA	1gyyB	1jfiA	1m1jF	1orgA	1r4vA	1td0A
1c3oD	1eizA	1h0mA	1jhgA	1m32F	1ou0A	1r5xB	1tggA
1c3yA	1ejxB	1h3fB	1jhnA	1m33A	1ou5B	1r76A	1tk1A
1c5aA	1ek5A	1h4dA	1jifB	1m6uA	1ox8B	1r8oA	1tk9D
1c6vX	1ekjA	1h6lA	1jk9B	1m93A	1oxzA	1r9qA	1tnbB
1c93A	1elqA	1h6wA	1jkzA	1mc9A	1oy5C	1rdi1	1tolA
1ccdA	1emnA	1h8uB	1jmtA	1mdaH	1p42A	1re0B	1trlB
1cl1A	1eniA	1hbkA	1jmwA	1mdaL	1p4uA	1ri6A	1ts3A
1cmxA	1eomA	1hfeS	1jpxG	1mhqB	1p59A	1rifA	1tsjA
1cokA	1eoqA	1hjdA	1jroA	1mjtB	1p6sA	1rjjB	1tv4A
1cpbA	1ep1A	1hkqA	1jtdB	1mmfA	1p89A	1rjlC	1txjA
1cq4B	1eptA	1hloA	1jw3A	1mmfM	1p8cF	1rkiB	1tyjA
1cqqA	1ergA	1hm0A	1jxhB	1mmoC	1p91B	1rmgA	1tz0C
1cr6B	1ex9A	1hmdA	1k0kA	1mmoG	1pbiB	1ro3A	1u10F
1csbA	1eyvB	1hpgA	1k12A	1mojD	1pjrA	1rq8A	1u2fA
1d1gB	1ezgB	1hpiA	1k3gA	1mqrA	1pjzA	1rrvB	1u6mD
1d1jA	1f0iA	1hpwA	1k3kA	1mt5P	1pkvA	1rsoC	1u6tA
1d1nA	1f1cA	1hqbA	1k42A	1mt6A	1pmjX	1ruwA	1u83A
1d1rA	1f3yA	1hqiA	1k49A	1mtlA	1pn3A	1rwrA	1u9hA
1d2iA	1f46B	1hsjA	1k6dB	1mwqA	1pnfA	1rybA	1ue9A
1d7rA	1f56A	1ht9A	1k85A	1mxfA	1ps2A	1rylB	1uf3A
1dbxB	1f81A	1huxB	1kcyA	1mzuA	1ps9A	1rz9A	1ufmA
1de2A	1fbqB	1hxmG	1kfrA	1n1fA	1pujA	1rzwA	1ughI
1de6D	1fdnA	1i4nA	1kmzA	1n4nA	1puzA	1s05A	1ugvA

1uh6A	1wgeA	1xvuA	2a8eA	2cdpD	2dvkA	2fgzA	2h7zA
1uhcA	1wgwA	1xvyA	2a8fA	2cdqA	2dwkA	2fh7A	2h8aA
1uhuA	1wixA	1y0eA	2a8lA	2ceoA	2dwvA	2firL	2hb0B
1uinA	1wj5A	1y10A	2adlA	2cg4A	2dxbC	2flfA	2hb6A
1ujvA	1wj6A	1y13C	2aehA	2cgqA	2dxlB	2fmmE	2hbgA
1um2A	1wjnA	1y1lD	2afbA	2cicA	2dy8A	2fmrA	2hdwA
1umgA	1wjrA	1y2xD	2ahvA	2cipA	2dyqA	2fmuA	2he3A
1umzA	1wjwA	1y47A	2aj7B	2ciqA	2e11B	2foiD	2hevF
1unnC	1wk1A	1y7nA	2akfC	2cjsA	2e44A	2fomA	2hf6A
1uocB	1wkcA	1ybfA	2araA	2ckiB	2e61A	2fprA	2hf7A
1usoB	1wm4A	1ychD	2arkF	2cm0A	2e6oA	2fqpA	2hihB
1uuzA	1wn0D	1yd6A	2au3A	2cmgA	2e7iA	2fqwA	2hj9A
1uwwA	1wnhA	1yhfA	2avnA	2cmpA	2e7kA	2ftuA	2hjdA
1uwyA	1wp8C	1yixB	2axkA	2cntD	2ea6A	2fv1B	2hjsA
1ux7A	1wpnA	1yk1B	2b0lC	2cofA	2ea9A	2fwtA	2hmfA
1uz5A	1wq2B	1yn3B	2b1rA	2couA	2eanA	2fwvA	2hntE
1v07A	1wt6D	1yn4A	2b2yC	2cq4A	2ebdB	2fx5A	2hqrB
1v2dA	1wtuA	1ynbA	2b34H	2cqvA	2ebjB	2fytA	2hrxA
1v5lA	1wvoA	1ypxA	2b42A	2crbA	2ebnA	2fz5A	2hryA
1v5rA	1wwlB	1yr0A	2b56A	2cswA	2ec3A	2g04F	2hszA
1v6zA	1wwtA	1ys3A	2b5dX	2cu1A	2ec6B	2g0fA	2ht1B
1v72A	1wx8A	1ysjB	2b61A	2cu5A	2ed4B	2g29A	2hu8A
1v9zB	1wz2B	1ysmA	2b6cA	2cunB	2ednA	2g2dA	2huoA
1vc4A	1wzzA	1ysxA	2b9dB	2cwrA	2edvA	2g2xA	2hvqA
1vcyA	1x19A	1yt8A	2basB	2cwzD	2ee1A	2g3kA	2hwtA
1ve3B	1x43A	1yu3A	2bb6A	2cx6A	2ee9A	2g4oA	2hx1A
1vehA	1x4xA	1yubA	2bcwC	2czcA	2eeeA	2g7lA	2hxdA
1veuA	1x5aA	1yxbH	2bfxC	2czsB	2eheA	2g9eA	2i0fE
1vffA	1x5pA	1yxeA	2bgwA	2d05A	2el5A	2g9iA	2i0kA
1vg0A	1x5yA	1yxyA	2bh1B	2d16A	2elaB	2gasB	2i0nA
1vi7A	1x86G	1z06A	2bh8B	2d2mD	2em3A	2gauA	2i25O
1vj1A	1x8dA	1z0kD	2bi0A	2d68B	2epmX	2gbbD	2i2wA
1vjfA	1x9iB	1z1nX	2bj1A	2d7rA	2eseA	2gbsA	2i3bA
1vkwA	1xa6A	1z5fA	2bkrA	2d8iA	2esvE	2gbzA	2i3eA
1vl4B	1xdyJ	1z66A	2bngC	2d9vA	2ettA	2ge5A	2i3oC
1vmdB	1xewX	1z6wA	2bq4B	2da0A	2etvB	2gftB	2i51B
1vnhA	1xf1B	1z7nD	2bswA	2dc4A	2evvA	2gjnA	2i5gB
1vq2A	1xfeA	1z85A	2btyC	2dceA	2ex5A	2gk1S	2i6tA
1vqtA	1xfjA	1za4A	2bugA	2dcpA	2exuA	2gk1T	2i6vA
1vs1A	1xg2B	1zbmA	2bv4B	2ddfA	2f1xB	2gqfA	2i7vA
1vyhA	1xg7B	1zclA	2bwbA	2ddwB	2f3lA	2gqiA	2ibbA
1vzmC	1xhoA	1zglU	2bwnA	2dfjB	2f3nA	2grjA	2ictA
1w2tF	1xhsA	1zgxB	2byoA	2dfxI	2f48A	2gt4C	2idgC
1w30B	1xi1B	1zi7A	2c1dB	2dgwA	2f4nA	2gujB	2if8B
1w57A	1xjtA	1zk8A	2c27A	2dj2A	2f5yA	2gvsA	2ifaA
1w63L	1xl8B	1zm8A	2c29F	2djwJ	2f6sA	2gypA	2ifgB
1w6vA	1xlrA	1zmbF	2c2iB	2dk3A	2f9cA	2gz4D	2ifrA
1wc2A	1xo1A	1zn6A	2c40B	2dkxA	2f9hB	2h2uB	2ifxB
1wckA	1xovA	1zvcA	2c45A	2dlbB	2f9iA	2h31A	2iiaA
1wd2A	1xppD	1zvfA	2c4fT	2dnnA	2fceA	2h32A	2ijcA
1wdeA	1xsqA	1zylA	2c4kF	2dqaA	2fenK	2h39A	2il4A
1wewA	1xsxB	1zyqA	2c9rA	2dr1B	2ffiA	2h3gX	2ilaA
1wf1A	1xt9A	1zzmA	2cakA	2drhA	2fg9A	2h3kA	2im9A
1wfkA	1xtzA	2a05A	2cb1A	2dryA	2fgeB	2h5gA	2imzA
1wfsA	1xv2D	2a3dA	2ccyA	2du6B	2fggA	2h62D	2inbA
1wfxA	1xvqA	2a6xA	2cdhZ	2dvhA	2fgrA	2h63D	2incC

2inpC	2khsB	2lyjA	2nzfA	2pijB	2r6iA	2vriA	2xc2A
2inyA	2khzB	2lzxA	2o14A	2pk3B	2r77A	2vrnB	2xdaA
2itbB	2kjzB	2m05A	2o2kB	2plhA	2r78D	2vs0B	2xdqA
2iu1A	2kknA	2m19A	2o4vC	2plnA	2rawB	2vseB	2xfoA
2iusA	2kl2A	2m1bA	2oaaB	2plyB	2rbdA	2vsqA	2xgnA
2iwkB	2koyA	2m2aA	2oajA	2plzA	2rdmC	2vtfA	2xgxA
2ixlB	2kp5A	2m3fA	2oasA	2pmrA	2rdnA	2vu8I	2xhfA
2ixoA	2kp8C	2m3lA	2ocaA	2pnyA	2re2B	2vxaL	2xhjA
2ixvA	2kqaA	2m72A	2ocxA	2pofB	2re3B	2vywA	2xj9A
2j1kC	2kqyA	2m7pA	2od2A	2ppeC	2rgkF	2vz8A	2xkhA
2j48A	2ks4A	2m98A	2odiB	2pqgB	2rh8A	2vzbD	2xm5A
2j6zA	2ksiA	2m9kB	2odkD	2pr7B	2ri9B	2vzwA	2xonL
2j85B	2ktmA	2mahA	2odlA	2prdA	2rjbD	2vzyA	2xp1A
2j9pA	2kuaA	2mbgA	2odxA	2prvB	2rpaA	2w1tB	2xqoA
2ja3F	2kuqA	2mdvB	2ofjA	2pvdA	2rppA	2w2eA	2xriA
2ja9A	2kw5A	2mf4A	2ogiA	2pviB	2rqeA	2w2mE	2xs6A
2jadA	2kw7A	2mj9A	2oh1D	2pyqD	2rr2A	2w38A	2xtlB
2jatB	2kwjA	2mjpA	2ohwB	2pytB	2rruA	2w4lA	2xtsD
2jb3B	2kxcA	2mk4A	2oiiA	2q1dX	2rspA	2w54A	2xxsA
2jbrA	2kxvA	2mk5A	2oikA	2q1fB	2rugA	2w68A	2xz8B
2jftA	2kzhA	2mklC	2olaA	2q1wC	2rukA	2w6rA	2y4oA
2jgvA	2l21A	2ml2A	2om6B	2q2cC	2sasA	2w8dA	2y9gA
2jhnB	2l57A	2mlzB	2oo3A	2q33A	2sqcB	2w8xB	2y9xH
2jjxC	2l60A	2mm8A	2opiA	2q42B	2u1aA	2w95C	2ybdA
2jksA	2l6zA	2mn4A	2oplB	2q4xB	2udpA	2wacB	2yc2A
2jmuA	2l73A	2mnaA	2oqgD	2q52B	2uydX	2wanA	2yewK
2jmvA	2l7mP	2mpbA	2or0B	2q74C	2uygL	2wb3A	2yg6A
2jp2A	2l7pA	2mswA	2oryB	2q7dB	2v0sA	2wcmA	2yg9A
2jp7A	2labA	2mt8A	2otjI	2q9uA	2v2fF	2wcvA	2yhnA
2jqxA	2lakA	2mteA	2ou2A	2qa2A	2v3qA	2we0A	2yijB
2jtcA	2lalD	2mu0A	2ouwB	2qb6A	2v5iA	2weuD	2yjkL
2jujA	2lb7A	2mv4A	2ov8A	2qc9A	2v6qA	2wjiA	2yjnB
2jwsA	2lbmA	2mvgA	2ovaA	2qckA	2v6vB	2wk8A	2ykoA
2jwuA	2lc2A	2mw0A	2owaB	2qe6A	2v73A	2wknH	2ylkA
2jxmB	2lepA	2mwm	2owhA	2qebA	2vduB	2wniA	2ylwA
2jz0A	2lfhB	A	2ox1A	2qffA	2vdvF	2wsiA	2ymsB
2jzjA	2lfkA	2mxzA	2oxhE	2qg6A	2verA	2wstA	2ymsC
2jzlA	2ljpA	2n1gA	2oy9B	2qgiA	2vfvA	2wt8D	2ymuA
2k0qA	2lktA	2n40A	2oysB	2qhtA	2vgyA	2wtoA	2yn2A
2k1pA	2lkzA	2n54B	2oz0A	2qiwB	2vh3B	2wukD	2ynmC
2k22A	2lllA	2n55A	2p0hA	2qjdB	2vh9A	2wuqA	2yo0A
2k2pA	2lm9A	2n87A	2p0nB	2qjxA	2vjeB	2wusS	2yp1D
2k3vA	2lnkC	2n8oA	2p1zA	2ql8B	2vkgA	2wvrB	2ypdA
2k4rA	2lnzB	2n8qA	2p25A	2qmmB	2vkiA	2wziB	2ypjA
2k5tA	2lojA	2nbtB	2p4zA	2qn5B	2vkvA	2wzlA	2yq7B
2k7lA	2lozA	2nc2A	2p5dA	2qneA	2vmeF	2x1jA	2yqgA
2k8hA	2lq6A	2nchA	2p5lH	2qnfB	2vmiA	2x28A	2yrnA
2k9xA	2lq9A	2nojB	2p6yA	2qqdC	2vmnA	2x2iB	2yscA
2kbxB	2lroA	2npnA	2p73A	2qryA	2vn4A	2x3bA	2yt8A
2kc7A	2lruA	2nq3A	2p7nA	2qsuB	2vneB	2x3hA	2ythA
2kcvA	2ls6A	2nrhB	2pb0A	2qu8A	2vnkD	2x5kO	2ytrA
2kd0A	2lstA	2nujA	2pblD	2qv8A	2vnrA	2x6nA	2yu2A
2kdwA	2lulA	2nvmA	2pcdE	2qvtA	2vo8A	2x7pA	2yv7A
2kepA	2lvqD	2nvwA	2pdoA	2qxxB	2vpsA	2x9iD	2yv9B
2kgjA	2lwaA	2nw0A	2pe4A	2qzgD	2vqaA	2x9jB	2yvhD
2kgxB	2lxmA	2nxnA	2pebB	2r15A	2vrcA	2xadA	2yvpA

2yw8A	3al6D	3c5oA	3dmyA	3f8kA	3godD	3hzeA	3k4zA
2ywjA	3al9B	3c5zA	3dnfA	3faxA	3gpkB	3hzrA	3k50A
2ywoA	3anoA	3c65A	3dnlA	3fc3A	3gr9H	3i0uB	3k7bB
2yxdA	3aqgB	3c6mD	3dohA	3fddA	3gs3A	3i3uF	3k7rC
2yz1A	3aw9A	3c6vC	3dplC	3fdhA	3gtzC	3i3vC	3k7tB
2yzoB	3axgO	3c7oA	3dtzE	3ff0A	3guzB	3i6qA	3k9aA
2z00A	3ay3D	3c7tD	3dwmB	3ff5B	3gv0A	3i70A	3kboA
2z1tA	3aygA	3c8lA	3dxiA	3ff8D	3gw4A	3i7jA	3kd9A
2z2uA	3azpA	3c8vA	3dxqA	3fg2P	3gw6A	3i7tA	3ke7A
2z3pB	3b0vC	3c9hB	3dzaD	3fg9F	3gw7A	3i8dC	3kesB
2z4fA	3b1bB	3cawA	3dztA	3fgcA	3gwqA	3i9kA	3kfvA
2z4iA	3b3cA	3cbpA	3dzuD	3fgeA	3gxgA	3i9sA	3ki0A
2z59A	3b48F	3ce9D	3dzvA	3fgyB	3gxrD	3ia0A	3kinD
2z5eB	3b49A	3cf4A	3e15A	3fhbA	3gyaA	3ia1B	3kkcD
2z5iJ	3b4qB	3cfsB	3e1tA	3fhgA	3gypA	3ic4A	3kkdC
2z5yA	3b59F	3cg1A	3e2jA	3fiiB	3gytA	3icpA	3km5B
2z6bA	3b5mD	3cjpB	3e4wA	3fj7A	3gyxB	3id9B	3km9X
2z6kB	3b5nK	3ckcB	3e6zX	3fkfA	3gyxC	3idfB	3kmhB
2z70A	3b5pA	3cmuA	3e8oA	3fljA	3h13A	3ie1D	3kmvA
2z8gA	3b5tA	3cooA	3e8xA	3fm3A	3h1nA	3ie2D	3kpaC
2z9zA	3b6aF	3coxA	3e99A	3fsdA	3h1qB	3igcA	3krnB
2za4B	3b7fA	3cpeA	3e9uA	3fveA	3h1tA	3ih5D	3ks6D
2zbaA	3b8oA	3cpxC	3ebnA	3fvwA	3h2iA	3ihwA	3kt9A
2zbmA	3b9zA	3cr8C	3ec1A	3fw6A	3h2vH	3iibA	3ktbD
2zc4E	3ba3B	3cspA	3ecqA	3fyaB	3h4qA	3iiuM	3ktdD
2zciD	3bacA	3csqD	3ed3A	3fz5D	3h4wA	3ilfA	3ktoC
2zdoA	3balD	3ctvA	3edyA	3fzgA	3h6eA	3ilhA	3kw3A
2zezA	3be6D	3cu2B	3ee6B	3g16B	3h7jA	3ilrA	3kxiA
2zgiD	3bfwC	3cuxA	3eetB	3g1cA	3h7zA	3io0A	3l15A
2zooA	3bg4D	3cvgD	3ef0A	3g1eB	3h87B	3ipjB	3l1pA
2zovA	3bgtA	3cwqA	3efaA	3g1oA	3h8sA	3irsC	3l2hD
2zozB	3bhgA	3cwvA	3efeA	3g23A	3h96A	3isuA	3l2nA
2zuyA	3bhwB	3cxpA	3efyB	3g7mA	3hc2A	3iu6A	3l4bC
2zwiA	3bijC	3cynC	3efzA	3g7pA	3hd6A	3iusB	3l5aA
2zwnA	3bj6A	3czxD	3eglC	3g7xB	3heyA	3iuwA	3l6aA
2zxiA	3bksA	3d3sD	3ej3B	3g8eB	3hf7A	3iwdD	3l8dA
2zzdJ	3bmaA	3d5pB	3ek9A	3g8wA	3hfnB	3iwtC	3l9tA
2zzeA	3bmzB	3d6nB	3elgA	3ga2A	3hhdB	3iz0C	3la2A
2zziB	3bogB	3d7jF	3enkA	3ga9S	3hj9B	3iz0D	3ladB
2zzkA	3bpbB	3d8kA	3eo4A	3gasF	3hjgA	3j31Q	3latB
3a0rA	3bq3A	3d8tB	3eo6A	3gb0A	3hkmC	3jcaC	3lccA
3a14A	3brnB	3danA	3eo7A	3gbrA	3hlzB	3jcmL	3lefA
3a23A	3brsA	3db0A	3eoiB	3gbvA	3hm5A	3jqxC	3lfkD
3a2bA	3brwA	3dc0A	3epnB	3gceA	3ho5B	3jrvB	3lgdA
3a2eD	3btzA	3dc7C	3eqtB	3gdmA	3hpdA	3jtwA	3li6A
3a4uB	3bwnA	3dciC	3excX	3gdtD	3hqiB	3ju0A	3lihA
3a4xB	3bytA	3dcpC	3exfA	3gewB	3hqvA	3jveA	3ll9B
3a54A	3byvA	3dddA	3exiA	3gffA	3hqvB	3jw8A	3llvA
3a76A	3bzbB	3dfiA	3exmA	3gfuD	3hrdF	3jwiB	3lm2A
3a9lB	3c01H	3dh0A	3exzE	3gg1B	3hrdH	3jxbD	3lmcA
3ab4O	3c02A	3dh8A	3ey7A	3gg6A	3ht2C	3jzzA	3lmeL
3ac9B	3c0qA	3di4A	3eypA	3ghdA	3hvvA	3k0zA	3ln6A
3agrA	3c1dA	3dlaD	3f13B	3gl5A	3hx8A	3k11A	3ln7A
3akaA	3c1uA	3dlwA	3f2gA	3gmgB	3hxkA	3k1jB	3lnbA
3akfA	3c4mD	3dm3C	3f7eB	3gmvX	3hyqA	3k20A	3looC
3akoB	3c5hA	3dmiA	3f8hA	3gmyB	3hzbA	3k25A	3louA

3lp9D
3lpeB
3lsrA
3ltgD
3lu2B
3lvuD
3lw9B
3lx7A
3lxxA
3lynA
3lyxB
3lzaA
3lzkD
3m05C
3m0nA
3m1gC
3m1uA
3m1yA
3m21F
3m5bA
3m6yD
3m7gA
3m9bA
3mb2A
3mb8B
3mcoB
3mczB
3mdpA
3me8B
3mepC
3mezC
3mhvA
3mipB
3mjkA
3mjtB
3mjvB
3mkyP
3mleA
3mmpC
3mpxA
3mqmB
3mruB
3msrA
3mvcB
3mvnA
3mw3A
3mx3B
3myfA
3myuA
3myxB
3mz1D
3n0aA
3n27C
3n2qA
3n3yD
3n6oA
3n71A
3n77A
3n9lA
3na0D
3nadA
3ndhB
3neyA
3njhA
3njxA
3nllA
3nnmB
3no4C
3no8A
3nokB
3nt6A
3ntdB
3ntsB
3nu7A
3nufB
3nuqA
3nv0B
3nvlB
3nvtA
3nwjA
3nybA
3nysA
3nzeA
3nzqB
3o5vA
3o66B
3o6pA
3o7kA
3oa1B
3oa5A
3oacC
3obeA
3obkA
3ocdA
3od9A
3oeiC
3oetF
3offA
3ofjA
3ogmP
3ogvA
3ogzA
3ohaA
3oj6D
3ol0A
3ombA
3omdB
3on1A
3onlB
3onoA
3op1C
3optA
3orcA
3orfA
3orjA
3os7A
3ougA
3oumA
3ovkD
3ow2G
3oweO
3owvA
3oy2A
3oyzA
3ozbF
3p04A
3p0cA
3p2uB
3p3vA
3p6yN
3p7jA
3p8cE
3p96A
3pamB
3pesB
3pf6D
3pftA
3pgbA
3ph9B
3phzA
3pijB
3pinB
3pisA
3pivA
3pjeB
3pjiA
3pjlA
3pjyB
3pkiA
3pm6B
3pmdA
3pqiA
3psjA
3psqB
3pstA
3pt3A
3pu5A
3pu6A
3pv2D
3pw9A
3pybC
3pz1B
3q09T
3q1jA
3q2sC
3q31A
3q34A
3q4rA
3q7tB
3q9cA
3qayD
3qb8B
3qbwA
3qc2A
3qcaD
3qfwB
3qh2D
3qheA
3qhpB
3qibD
3qiuC
3qmmA
3qocD
3qp6A
3qphA
3qqmA
3qr2A
3qr8A
3qrhA
3qrxB
3qryB
3qtgB
3qvaA
3qvoA
3qw4B
3qy3A
3qyqA
3r03A
3r07A
3r0lA
3r0xA
3r2xC
3r5eA
3r6fA
3r74A
3r7wD
3r89B
3rbzA
3re2A
3renA
3rfiA
3rfqC
3rfxC
3rfyA
3rioA
3rj5A
3rm5B
3robD
3rpeB
3rpfB
3rpwA
3rrwB
3ru8X
3ry0B
3rzvA
3s0gB
3s0tA
3s2rB
3s6dA
3s6oD
3s6sB
3s9fA
3sajD
3sb3A
3sdxC
3sf5C
3shfA
3shvB
3snxA
3solA
3somA
3sotF
3sp1A
3spsF
3sq3D
3st1A
3stqA
3sxqB
3sxyB
3t12A
3t38A
3t46A
3t4tB
3t61B
3t6oA
3t6rB
3tbjA
3tbmA
3tcjA
3td7A
3tdmD
3tdvA
3tf4A
3tghA
3tm1A
3tm9A
3tmdA
3to4D
3tosJ
3trjD
3tsnA
3tspA
3tssA
3ttgA
3tuaA
3tyjA
3tykA
3tyrA
3tzkB
3u0jB
3u22B
3u4gA
3u4yB
3u7vA
3u7zB
3u9eA
3ua3A
3uatA
3ub0A
3ue5B
3ugkA
3uk8B
3ulyB
3umvA
3un9A
3uotA
3upuA
3upyA
3ustA
3utoB
3uunA
3v2gA
3v7iA
3v93A
3vadA
3vcxB
3vezA
3vj7A
3vjzA
3vkdB
3vnnA
3vocA
3vpcD
3vs8H
3vs9E
3vsvD
3vtiC
3vtnA
3vuoA
3vvlB
3vx8A
3vyiA
3vzqA
3w0rA
3w1vB
3w1yB
3w9eC
3waqA
3wb9A
3wdoA
3wdyA
3we9A
3wflA
3wh9A
3wj7A
3wlaC
3wn2A
3wobA
3wqoA
3wqyB
3wr2F
3wsbA
3wtrA
3wucB
3wv9D
3wvcB
3wy8A
3x0fA
3x0tB
3x2oA
3zbgB
3zcbB
3zh0D
3zi1A
3zj0A
3zk0A
3zk4C
3zm8A
3zmnA
3zn6B
3zpvY
3zr6A
3zt9A
3zueB
3zusA
3zv0B
3zw5A
3zxbD
3zxqA
3zxsC
3zy6A
3zymA
3zytA
3zzlC
4a0eB
4a1mA
4a1rD
4a2wB
4a47A
4a5kA
4a6aA
4a8xC
4a91A
4aayB
4ab7H
4abmA
4abtB
4ad9F
4adxI
4ae8D
4ag9B
4agvD
4ai5A
4aitA
4ak1A
4akmA
4aktA
4alfB
4am9A
4ao8A
4asmB
4aupA
4av8A
4avaA
4ay8B
4azvA
4azwA
4azzA
4b0iA
4b1wM
4b2zA
4b6uA
4b8bB
4b8nD
4b96A
4b9jA
4bacB
4bbkA
4bc5C
4bduA
4bg8A
4bi9D
4bitA
4bjhB
4bk2A
4bmkA
4bmyA
4bn6A
4bndB
4bnqA
4bofH
4bosB
4bqlA
4bqoB
4bt2A
4bugB
4bvlD
4bwhA
4bwoB
4bwpB
4bwxB
4bxlA
4bz0A
4c1bC
4c1rA
4c2dD
4c2eB
4c2gA

4c5nD	4eijA	4gjjD	4im9C	4lc9A	4nknA	4pmrA	4rbrB
4ccyA	4eivB	4gkpA	4in0B	4le5B	4nocA	4pmyA	4rbwD
4cdzA	4ejzB	4gmgA	4ip6A	4leeD	4nohB	4pnhL	4rg8A
4ce7C	4eoyA	4gmjE	4ipbB	4lfnC	4nqwA	4pprA	4rjvD
4cgeF	4eozA	4gn2A	4ipvB	4lgiA	4nrhA	4pq1A	4rkzB
4cgxA	4epqA	4gp0A	4itrB	4liyA	4nrnA	4pu3A	4rmbB
4chxB	4eqaB	4gp6A	4ivnA	4lj4A	4ns5A	4pu6A	4rn3B
4ci3B	4etqX	4gpsA	4iw7A	4lm9A	4ntgA	4pvcA	4ro5A
4ci6B	4eueA	4gq1A	4ix1A	4lmsC	4ntlA	4pzvA	4rpcB
4cjcD	4evmA	4gr2A	4ixsA	4lowB	4ntwB	4q05B	4rrgD
4cknD	4evuB	4grfA	4iypC	4lp7A	4nv1C	4q2jA	4rs2B
4cktB	4ewgA	4guaA	4izeA	4ls3B	4nvsA	4q2wA	4rsjD
4clvB	4ewlB	4gucB	4j2cC	4lsyB	4nw4A	4q3oA	4rt5B
4cngB	4exbF	4gveA	4j2pA	4lv5A	4nx8B	4q56A	4ru4B
4cooA	4eywA	4gwmB	4j2sA	4lv8A	4o5sB	4q5oB	4rv9A
4cpgB	4f03A	4gyoB	4j3qA	4lw8A	4o6gA	4q5wB	4ryiA
4cq4A	4f0yB	4gyrA	4j41A	4lwkB	4o8uF	4q69B	4rz0A
4cu9A	4f1iA	4gz9A	4j4sA	4m01D	4o9uB	4q6qA	4rzhA
4cxkB	4f3rA	4h0aA	4j7pA	4m1lB	4oaeA	4q6xA	4rzuB
4cyjD	4f4oC	4h2dA	4j8lA	4m1xD	4obbB	4q82B	4s1mB
4cz4A	4f69A	4h9kA	4j8pA	4m73B	4obiA	4qafB	4s2rP
4czyC	4f72B	4hciB	4jbeB	4m8dL	4ocoA	4qakB	4s37A
4d1fA	4fb3E	4hdkB	4jd0A	4mb8D	4ofqA	4qaqA	4s3nA
4d3lA	4fb9A	4hedA	4jenC	4mctD	4ohfD	4qayA	4tkzF
4d59A	4fbmA	4hesE	4jgjB	4mdoA	4oi6B	4qccB	4tlmD
4d77A	4fd7D	4hfoA	4jobA	4me3A	4oirA	4qdkB	4tlxA
4d7pA	4fe9A	4hfsB	4jpdA	4mgfB	4ojvA	4qeyJ	4tn5A
4dayA	4fetA	4hg2B	4jqsC	4mixA	4ok7C	4qhjB	4tnmA
4dddA	4ffrA	4hheA	4js1A	4mjsA	4okqB	4qhxA	4tqmA
4dgfB	4fgcA	4hlyB	4jupA	4mkyA	4onxF	4qhzD	4tr5A
4dghB	4fgiH	4hqlA	4jxbA	4mn0A	4oogC	4qkfC	4tt9D
4dh2A	4fioC	4hr6B	4k00A	4mn7A	4oouB	4qkuA	4tvsA
4di3D	4fmaA	4hrfA	4k05B	4mniA	4opuA	4qprA	4tweA
4dl1A	4fshA	4hrgB	4k28B	4mnqD	4oq1A	4qqrB	4tyzB
4dmgB	4ftwA	4hrwB	4k2xB	4mowD	4orlA	4qs9A	4u10B
4dmuK	4furA	4htfB	4k6bC	4mr0A	4owzB	4qsfA	4u2eA
4dooA	4fyuC	4htiA	4k80A	4msxA	4oyuA	4qtpD	4u3cF
4dryA	4fzxC	4hu2A	4ka4E	4mtnA	4p0yA	4qtuC	4u3sB
4ds2A	4g0sB	4huzA	4kjmA	4mwaA	4p1bI	4qtzA	4u49A
4dwnB	4g3aB	4hvmA	4kngE	4mx2A	4p1zD	4qwoB	4u4eA
4dzaA	4g3tA	4hx3A	4knsA	4mz0B	4p2bA	4qyzK	4u4jA
4dzgA	4g3vA	4hx3L	4kqdD	4mzqL	4p2cF	4r04A	4u5hH
4dzpB	4g4fA	4hy3D	4ksmA	4n0gC	4p2qD	4r0lC	4u6bA
4e0qB	4g4jA	4hzpA	4kzkA	4n11A	4p4nC	4r0tB	4u6dB
4e1lD	4g76A	4i0nA	4l22A	4n2rA	4p5nB	4r0zA	4u77A
4e27A	4g7wC	4i0wC	4l3fH	4n4gA	4p6vA	4r1tA	4u7jA
4e2gA	4g8eA	4i1dA	4l4uA	4n4hA	4p94B	4r27A	4uc4A
4e61A	4g9iE	4i43A	4l58A	4n6bF	4p9uE	4r2fA	4ue5D
4e89A	4ga0A	4i5lB	4l5nF	4n80A	4pagA	4r2mB	4ufcB
4ea4A	4ga5B	4i69C	4l5tA	4n82B	4pawA	4r3vA	4ufqB
4ecgA	4gbmA	4i6kA	4l63B	4n9xA	4pc4A	4r42A	4uhqB
4edlD	4ge0A	4ibnA	4l7vA	4nfcA	4pdtA	4r72A	4ulwA
4edpA	4ge1A	4icyA	4l8fA	4nfyB	4ph8B	4r7xA	4um5A
4eeeB	4gebB	4ifeA	4l8oA	4ng0C	4piwA	4r81D	4umwA
4ei9B	4ggzD	4iknA	4l9aB	4ni3A	4pkxA	4r8xD	4unmA
4eibB	4gigA	4ilvA	4lb7B	4nk2B	4pllB	4r9iA	4unnA

4upgA	4xpkA	4zuyB	5c2iA	5eayA	5h6oA	5jp9B	5lcnA
4urjD	4xq1A	4zv4C	5c2wF	5ecfA	5h8zA	5jqfB	5lefD
4urpA	4xqfA	4zv9A	5c4rA	5ee2A	5h9mB	5jseA	5lg4A
4urrF	4xqjA	4zvdB	5c9iD	5efrA	5hbqA	5jtaA	5lizA
4ushA	4xrrB	5a29A	5ccoA	5eghB	5hdlA	5jtnF	5ljoC
4uurA	4xsgB	5a3kA	5cd7F	5eipB	5hdpG	5jtoE	5llkA
4uw0A	4xsqB	5a53A	5cerA	5ek8A	5hk0D	5jtqE	5llxB
4uwqA	4xuvA	5a60A	5cftA	5eovA	5hm9A	5jvvA	5ln4A
4uwqB	4xwtA	5a7mB	5cgnE	5epqA	5hnmA	5jw7B	5ln5A
4uy4A	4xxhB	5a7rA	5chhA	5eq4A	5hp5A	5jwcH	5lo9B
4uytA	4xywA	5a96A	5cj9A	5eqvA	5ht6B	5jwfA	5lorA
4uzkB	4xzvC	5a9cA	5cjjB	5esuD	5hx0B	5jwgB	5lphA
4v07A	4xzvH	5aa7B	5cjpE	5ewmD	5hxbX	5jxfD	5lq7A
4v0hD	4y06B	5abbZ	5cnlB	5exkK	5hy4H	5jysA	5lrqA
4v14A	4y1eA	5aeoA	5cu1A	5ey7A	5i14B	5jzgC	5lu3A
4v1rA	4y1qA	5ag8B	5cupB	5f5vD	5i2cA	5jzxF	5lw8A
4v2pA	4y4nC	5ah5B	5cv1A	5f67B	5i33A	5k0wB	5lxuA
4v33B	4ycrA	5ahuA	5cwmA	5f87F	5i47B	5k2mA	5lxvA
4v3dD	4ycsA	5aj2C	5cx7P	5f8cA	5i5hA	5k2mE	5m1qA
4w2rA	4yeqU	5aj9A	5cx8A	5fb1A	5i5oB	5k35A	5m2sA
4w66A	4yg6D	5ak8A	5cxlB	5fdkA	5i62A	5k5fA	5m2sB
4w78G	4yhcA	5aohB	5cyjB	5fldA	5iebA	5k5uA	5m3iA
4w7oA	4ykaD	5ap8C	5czyA	5fliA	5if6F	5k5vA	5m88A
4wcjA	4ympC	5avmA	5d1lB	5fmf1	5ihvA	5k6dB	5m8pA
4wgkA	4yn2A	5avqA	5d2iA	5fmtA	5ijoX	5k6fF	5manB
4wh0H	4ynhB	5awfB	5d5nB	5fq6C	5ijxA	5k8gA	5mehA
4wiaA	4yo1A	5awqA	5d5pD	5fsbA	5ikdA	5k8kA	5mg5X
4wiwF	4yrhB	5axhA	5d5zE	5ftyA	5ikyA	5kbkA	5mgyF
4wj2A	4yryA	5azcA	5d7bB	5fu4B	5il5B	5kbmA	5mh2A
4wjlB	4yteA	5azxA	5ddtA	5fukB	5im6A	5kc2B	5mh4A
4wkwB	4ywaD	5b0pA	5detB	5fuxB	5imkA	5kc9C	5mj7B
4wliA	4yxfB	5b0vA	5dgrB	5fwsA	5ipuB	5kcnA	5mk5D
4wmoA	4yxzA	5b37F	5dhmC	5fxpB	5it0A	5kf1A	5mlfF
4wneB	4yypB	5b4pC	5diyA	5fydB	5it3A	5kh6A	5mpvD
4wopA	4yzoA	5b5uB	5dj5B	5fyoA	5itmF	5khuI	5mr6G
4wqsC	4z02A	5b5xA	5djkA	5g0fA	5ityC	5kiyB	5mtcA
4wsgA	4z45A	5bjwB	5dl8A	5g52C	5ixuA	5klfA	5muiA
4wtsA	4z48A	5bkmA	5dljF	5g56A	5izwA	5kniA	5mv0D
4wx3A	4z4mA	5bobE	5dlyA	5g5hB	5j0cB	5kp8B	5mv9B
4wx7C	4z7rA	5bp7C	5dmpA	5ggfA	5j1dA	5kshB	5mz2I
4wy2A	4z80A	5bp8A	5dmrA	5gqhC	5j1tA	5kuaA	5n35A
4x0gD	4z85A	5bptA	5dniA	5graB	5j4fB	5kuhA	5n4kB
4x28C	4z9nA	5btwA	5drkA	5groB	5j4mC	5kuyI	5n6mA
4x2xA	4za3B	5bu1A	5dwcA	5gt5B	5j60A	5l0pA	5nb1F
4x36A	4za6B	5bu2A	5dwzG	5gteA	5j7cD	5l16A	5ncsA
4xa9a	4zciA	5butA	5dx5B	5gx6A	5jbtY	5l2eC	5ngnC
4xc8A	4zdeC	5buvA	5dy9A	5gz8A	5jcdC	5l2lF	5nh2B
4xc9A	4zhtD	5bxfA	5dzgA	5h0iB	5jd9A	5l33A	5nl1A
4xchA	4zjmA	5bxxA	5e4eA	5h1nB	5jdoB	5l37E	5nm8B
4xcmB	4zkdA	5bxyB	5e4mB	5h3dD	5jfzA	5l6mA	5nmzD
4xf5B	4zm6B	5by2A	5e50B	5h40B	5jg7B	5l7vB	5nnlB
4xg0A	4znbB	5byoA	5e59A	5h45B	5jg8A	5l81A	5ntbB
4xhfA	4znyA	5bz4M	5e5bA	5h4bA	5jh5A	5l8jB	5nw4h
4xi1C	4zosD	5c00D	5e5yD	5h4uC	5ji5A	5l9sA	5nx3D
4xj6A	4zq8A	5c0oH	5e6tA	5h5oB	5jk4A	5la4A	5o0xA
4xjcA	4zquB	5c1iD	5e8bA	5h67A	5jmfA	5lalA	5o1mB

5o4hA	5twaB	5vnzD	5xvlA	5z75D	6avyA	6d6vH	6f9fI
5o5oD	5txtF	5vo5A	5xvsB	5z7gA	6b2eA	6d7aA	6fbzB
5o63B	5u25A	5volA	5xxaB	5z7iA	6b2eB	6d7kF	6fc2D
5o9yA	5u2pA	5vqjA	5xyiU	5z96D	6b2xA	6danA	6fc5A
5o9zz	5u4qB	5vrdD	5xzkA	5zauB	6b8dA	6daoB	6fcxA
5oa9A	5u56B	5vtsA	5y18B	5zbtA	6bdjU	6dbiD	6fejA
5ocrD	5u5iB	5vttA	5y28A	5zdeC	6bevB	6dftI	6ff70
5odjA	5u75A	5vxyA	5y2vA	5zecA	6bitH	6dg3L	6fhpA
5odqG	5u7nA	5vyeA	5y39F	5zedA	6bk5A	6dj8A	6fjuA
5oenA	5u9nB	5w0aB	5y4cA	5zewA	6bm7C	6dkuA	6fljA
5of2A	5u9zB	5w1nD	5y4kA	5ziqD	6bmcA	6dmqA	6fn6A
5ofyA	5ua4A	5w3gA	5y6lA	5zizA	6bp8A	6dnuB	6fnzD
5ofzD	5uasA	5w4aD	5y6lB	5zjbA	6bphA	6do3A	6fpcD
5ogxA	5ub7B	5w5mB	5y6oI	5zjkA	6bprA	6dqoA	6fprB
5oj3A	5ublA	5w6lB	5y7dA	5zjlA	6brmH	6dr3A	6fq0B
5ojyA	5ucgA	5w7kA	5y9hC	5zkhB	6bs5A	6drmA	6fskA
5okuA	5uddH	5wa3A	5y9yA	5zl6A	6bswB	6drqA	6fv3D
5ol0A	5ue2A	5watB	5yb8D	5zmpA	6btmA	6dt1E	6fw4A
5ol8D	5ue6I	5wdxA	5yc4A	5zokD	6bvvB	6dx5A	6fwsB
5oljA	5ufmA	5wecB	5yc9B	5zolA	6bwcF	6dy2C	6fxaB
5olqA	5ufvA	5weeD	5ydcA	5zq5C	6bxtC	6dzkY	6g13A
5oltA	5ug7A	5wgvA	5ydfA	5zqvH	6bzxA	6e0lA	6g1nA
5oluA	5ugzB	5whkA	5ydgA	5zruC	6c0dA	6e0mA	6g1oA
5onuA	5uidD	5widC	5ygrA	5ztpA	6c29A	6e14D	6g39A
5oo8A	5uk5B	5wkhI	5yk3B	5zw9A	6c2vA	6e1qA	6g3aA
5oq2A	5um7B	5wlkD	5yk5C	5zz7B	6c4mC	6e33A	6g45C
5ovjA	5umqA	5wlnN	5yk5D	5zzbA	6c4uB	6e4qA	6g4hA
5ovnA	5uohA	5wo3B	5ynrA	5zzzA	6c8sB	6e5uA	6g4wq
5ow0B	5upbD	5wp5B	5yp7D	6a55A	6camA	6e6aA	6g6lG
5ow2B	5ux5C	5wtfB	5yqqA	6a56A	6canB	6e6sB	6g70B
5oxwD	5uxbB	5wufA	5yr0A	6a5bA	6cb1A	6ebyB	6g8rB
5oxxB	5uxhA	5wx1A	5yriB	6a5eD	6cb6A	6ec3C	6g8wA
5suhA	5uz9K	5wxkB	5ysqB	6a6cA	6cblH	6edxA	6gaoC
5t17A	5v0fA	5wyaA	5ytiA	6a6gD	6cboD	6efwA	6gcvC
5t1jB	5v0lA	5x14A	5yufD	6a6oA	6cd2B	6eg0B	6gdxC
5t1pH	5v1aB	5x3hB	5ywzA	6a6yB	6cgoA	6ehcB	6gemA
5t2fA	5v1uA	5x4aA	5yxiA	6a83A	6ch3B	6ejfP	6ggkB
5t3pC	5v1xH	5x56A	5yy2A	6a8wA	6chsA	6ejvB	6ghrE
5t3wA	5v36B	5x8zA	5yzmC	6a8xB	6chsH	6ekmB	6ghsA
5t4xA	5v47B	5xaqB	5z0zA	6a93A	6cj6A	6el3A	6giqf
5t6oA	5v4aB	5xc2B	5z25A	6aa9A	6ck0A	6emkC	6gmaA
5t88A	5v5qA	5xdiA	5z28A	6ab6C	6ckqA	6enkA	6gp5A
5tckA	5v6bA	5xe2A	5z2xA	6af4H	6cnoD	6epfW	6gpaB
5teaF	5v6iA	5xf7A	5z34A	6agmA	6cnuA	6erpF	6gpmA
5tfoB	5v8cA	5xfhA	5z3cA	6ahuC	6conB	6et8B	6gq4A
5tfzA	5v8sB	5xfmA	5z43A	6aiiA	6cpjA	6et9I	6gqeA
5tizA	5va2A	5xg5A	5z4gA	6ajqA	6cptA	6eu6A	6gqfD
5tooA	5vapB	5xgpA	5z4tB	6akjA	6cqnE	6euaC	6gqnB
5towB	5vbbA	5xkoA	5z51B	6aklA	6crdH	6euiA	6gtaA
5tprA	5ve9A	5xm3B	5z62D	6am0A	6cunA	6ewgA	6guiB
5tpvA	5vebY	5xoiA	5z62I	6apjB	6cuqC	6ewpA	6gw6A
5tpwA	5vhfd	5xr2H	5z62L	6apnA	6cvqA	6exfD	6gybS
5tqmA	5viaA	5xr3H	5z62M	6aq3A	6cw9D	6exuA	6gzcC
5ts4D	5vj7B	5xtkA	5z6eA	6asdC	6cyzA	6ezmX	6h0jA
5tsyA	5vl3Q	5xumA	5z6pB	6au1B	6czmF	6f2rQ	6h1qB
5tvmF	5vlxA	5xuoA	5z70A	6avgB	6d2zA	6f74D	6h1xA

6h2bC	6j6aB	6kupA	6mltA	6otvB	6r46A	6tdwB	6v6nA
6h3jB	6j7cA	6kvxA	6mlyA	6ovkR	6r4lA	6tedX	6v7nB
6h4dA	6j7nA	6kw6A	6mmxD	6owdB	6r5kA	6tejA	6vbnA
6h5hA	6j7xA	6kxdB	6mn5F	6owrA	6r5kH	6tfoC	6vcdA
6h5lB	6j8oB	6kxfA	6molA	6owtA	6r5kN	6tg2A	6vdeA
6h60A	6j9cD	6kxsA	6mpaB	6oxmB	6r5kO	6tgtA	6ve1A
6h9eB	6jbjB	6kxuH	6mr1A	6p18P	6r5mA	6thmE	6vfsE
6ha7D	6jcnB	6kyuB	6mr3C	6p2aF	6r60A	6thtA	6vggD
6hbeC	6jd2D	6l0uB	6mtqT	6p3qB	6r7hF	6tj2A	6vgvA
6hceA	6jdkA	6l0vG	6mw8A	6p74A	6r8iA	6tj5B	6vjaC
6hciA	6jhpD	6l1eA	6my0A	6p8uA	6r9vA	6tjiB	6vjiA
6hf1A	6jk7A	6l1rA	6mzoA	6pa7N	6rc9A	6tjlB	6vjpB
6hftA	6jkuD	6l2uB	6n7rC	6palA	6rfuB	6tk0X	6vjrC
6hhbD	6jl0A	6l42A	6n7xC	6pbcA	6rj0A	6tn6A	6vkzA
6hjmA	6jm5A	6l5hA	6n9qP	6pbzA	6rjkA	6tp5A	6vp8B
6hosA	6jmgA	6l6jA	6nagB	6pkuD	6rlaA	6tpiA	6vptA
6hqjA	6jmtF	6l7mA	6nb4C	6psfA	6rmnB	6tpqA	6vq5A
6hqvB	6jq1B	6l7oI	6nbgF	6ptkD	6rreF	6tqhA	6vs4B
6hs7e	6jqdA	6l86A	6nbrD	6puaA	6rs2B	6trmA	6vshC
6htoB	6jqzA	6l8eA	6ndxB	6pwjA	6rs9A	6ts8A	6vsjA
6htvA	6js9A	6l9sA	6njnA	6px6D	6rtqB	6tutR	6vt5A
6hvgA	6jt6A	6lb9A	6nkiA	6q01D	6rxsA	6tv6A	6vxkB
6hwlB	6jtuA	6lcnF	6nluB	6q09A	6s1aB	6tv9H	6vxwA
6hx9A	6jucA	6leeA	6no4B	6q22B	6s1kB	6tylC	6vy0D
6hzfB	6jzlA	6lfsA	6nobA	6q41C	6s1zA	6tzwB	6w32C
6i0aA	6k0aG	6lhnA	6nowA	6q4wB	6s4cA	6u05A	6w3oA
6i0xB	6k0iA	6lk4A	6nppB	6q5sA	6s5eA	6u2pA	6w66B
6i1qA	6k1eB	6ll8B	6nq4A	6q63B	6s5jA	6u3vC	6w9rL
6i2nD	6k1fB	6lp4A	6nqiA	6q8xX	6s7qH	6u51B	6weo2
6i2vA	6k1yA	6lpaA	6nryA	6qaiA	6s7tH	6u83A	6wf4A
6i2zB	6k2fA	6lqeA	6nsrM	6qajA	6s9rA	6u8yl	6wfqC
6i50A	6k4jA	6lqgA	6nt9A	6qayA	6s9uA	6u8yN	6wg9A
6i56E	6k4yM	6lrhA	6nu7A	6qciD	6sc9A	6uadA	6wgyA
6i57A	6k5gA	6lvnD	6nx5D	6qdlA	6sdfA	6uanB	6wigA
6i5bB	6k7fA	6lw3A	6ny0A	6qe3A	6sduD	6ubbD	6wjaB
6i97A	6k95C	6lw4A	6ny5B	6qe6A	6sjiA	6uekA	6wjbB
6iaaC	6k9nD	6lxdB	6ny9B	6qekB	6sjoB	6ug5K	6wjgD
6ic4I	6ka3D	6lxwS	6nyrA	6qfoA	6skaD	6uh4D	6wpuA
6iciA	6kacP	6m13b	6nzdG	6qg0I	6sqgA	6uh5L	6wqbA
6ierA	6kacQ	6m2aA	6o2uA	6qgtG	6sslB	6uj5B	6wqiB
6iheB	6kd6D	6m2hB	6o3eA	6qh4D	6steC	6ukeX	6wqpB
6iiwA	6keaD	6m4lA	6o6bD	6qigA	6suaB	6unfB	6wqxD
6ijeA	6kgfA	6m7zA	6o6lA	6qilD	6sv3A	6ur0A	6wt6A
6ilxA	6kgfB	6m8mA	6o7cA	6qj6E	6svkA	6urfA	6wt9A
6iqsD	6kgjA	6m8tA	6ocgB	6qkxA	6sxjA	6ut6A	6wtiB
6ir8A	6ki3B	6m9cA	6oddB	6qp4A	6sy9A	6utsA	6wueB
6islB	6ki9A	6m9yA	6oewB	6qpjA	6t0vC	6uv7B	6wvsA
6iuqB	6kmaB	6mapB	6ohlA	6qpkB	6t1dF	6uv8A	6wwdA
6ivdB	6kmfA	6mc8B	6oitB	6qveW	6t3iA	6uwtH	6wxvA
6ixhO	6kodA	6mf4A	6onnB	6qwlQ	6t70A	6ux2A	6x0aa
6iy6E	6koeG	6mfvA	6onpA	6qwoA	6t7eA	6uxgC	6x0aA
6j0eA	6kp1B	6mgdA	6opfF	6qzuA	6tadA	6uxuB	6x1oB
6j1iA	6kqcA	6mgnA	6oplA	6r17C	6taqB	6v4oW	6x6oA
6j2uA	6kr6A	6mi5X	6ormC	6r1bD	6tb2E	6v4xH	6x8nA
6j38A	6krjA	6mj8B	6os3A	6r1gA	6td6B	6v55A	6x91H
6j3aA	6ksyA	6mlmA	6otdA	6r2hA	6td7A	6v67A	6xb5A

6xfkA	6zy0C	7cjxA	7ep2B	7l0jA	7o3iA	7sh3B
6xh9A	6zymB	7ckaA	7ep9G	7l1fD	7o78A	7shgA
6xi9B	6zzqA	7cleA	7epqB	7l4aA	7o79A	7sj9N
6xjhC	7a17C	7cljA	7epuB	7l5mB	7o9mM	7sp5A
6xkbE	7a4pC	7cm1B	7essA	7l6wA	7obqz	7sr2B
6xkwp	7abh7	7cneA	7eu34	7l78B	7ociC	7stdC
6xmiF	7adyF	7co1F	7evrC	7ljtB	7od2A	7sv6A
6xnsA	7afuA	7cogD	7exkF	7lkjA	7odtA	7sybA
6xssA	7aglA	7cp6A	7f21B	7lpoC	7ofqr	7t2yB
6xt4A	7ahhD	7cpxA	7f2yA	7lr1A	7ogtB	7t30B
6xy1A	7amvW	7cqsA	7f5iA	7ltsA	7oi6z	7t3bE
6xycA	7aoaD	7csjB	7f6pA	7lv8D	7okxM	7t4rA
6xydD	7aseA	7csqB	7f6tA	7lvtD	7okyZ	7t71A
6xz6D	7auuA	7ctqA	7f6uA	7lxcA	7om8Y	7t8wD
6y11O	7awvA	7cueH	7f6xB	7ly7B	7omcD	7tacA
6y3zA	7axzB	7cuzD	7f7qA	7m0iF	7oqhF	7tbsA
6y45A	7b1gD	7cw1B	7f8aA	7m6uD	7osbC	7tfmA
6y4fA	7b1sB	7cwhB	7fdeP	7m7fA	7oskB	7thhA
6y4rA	7b2cD	7cwqA	7fevA	7mbnD	7ot8B	7tlx1
6y6kA	7b2cF	7cx1C	7ffpA	7mcdD	7oxgB	7tmvB
6y87D	7b2oA	7cxvB	7fisD	7misC	7oywA	7trwA
6yagD	7b2sA	7czhB	7jhiA	7mpyA	7p0lA	7txmA
6yahB	7b2xA	7d1iA	7jicA	7mqsH	7p3bB	7ughB
6yczC	7b5lT	7d5cA	7jizA	7mqyA	7p9qF	7v6dA
6ygaA	7b7hA	7d6mB	7jj9A	7mskA	7pagA	7v6iA
6yifC	7bczD	7d89A	7jluB	7mwcA	7pc6H	7vdvA
6yj1A	7bfyA	7d9iA	7jrjK	7mwrB	7pknM	7vgmA
6yleA	7bjcB	7dbwC	7jrpg	7mwzD	7ppcL	7vi7B
6ymyk	7bkbJ	7dc4B	7jt5A	7myvA	7pq8A	7vw1A
6yp4A	7bobC	7dl0B	7jthH	7n09A	7prrB	7w2vA
6yq4C	7bqlB	7dl1C	7jtkJ	7n1nA	7q1bA	7w5vA
6ys3z	7br1A	7dl3A	7jtqC	7n7iC	7q3lq	7wkkj
6ysnA	7bv6C	7dnmA	7k0qA	7n7sF	7q5bD	7x0dA
6yv7B	7bv6V	7dnpA	7k0zC	7n8oQ	7q6zB	7xayA
6yy6A	7bvzA	7dpaD	7k34D	7nb0A	7qbkA	
6z05L	7bwnO	7dskA	7k3pB	7ncyA	7qilA	
6z06A	7bwyC	7dveA	7k5cD	7nczA	7qroA	
6z0oF	7byyD	7e0lA	7k67A	7nd0C	7qs4D	
6z1hA	7c03A	7e2iG	7k7jA	7nekA	7qs5B	
6z2iA	7c13C	7e2rB	7kb2B	7nh7B	7r65A	
6z31B	7c1eA	7e36A	7kflB	7njnN	7r6oA	
6z82A	7c1iF	7e37A	7kfuB	7nljB	7r76A	
6z9lA	7c3vA	7e4lB	7kfuD	7nmmA	7r7bA	
6z9uA	7c5zB	7e4mA	7kjnA	7nmqA	7rdrA	
6zb8B	7c6oA	7e62C	7kn1A	7nmsA	7reaA	
6zc1A	7c7dA	7e9dA	7kpzA	7nn6A	7reiA	
6zejO	7c7sA	7ebkA	7ks3C	7np8A	7rg7A	
6zfcF	7c7uA	7ecqA	7ksbA	7npaP	7rg8A	
6zi8A	7c8fB	7eeiA	7ksnA	7nq4B	7ri3D	
6zmwL	7c8gB	7eevA	7ksoF	7ntgA	7rpqB	
6zplC	7caqA	7eftA	7kw6A	7nuuB	7rrmC	
6zsoA	7cdvB	7eg5B	7kwsA	7nwfA	7rztC	
6zv9B	7cfeA	7eg7c	7kwtB	7nyrG	7s63D	
6zw9A	7chuA	7eg9B	7kxyB	7o2gA	7scfA	
6zxwG	7cigA	7el1A	7l00D	7o37k	7sciA	
6zxwH	7cj9A	7ennK	7l0iA	7o3eI	7seuA	
