# Supplementary material for: Automated Protein Secondary Structure Assignment from Cα Positions Using Neural Networks
Source: Biomolecules. 2022 Jun 17;12(6):841. doi: 10.3390/biom12060841 (PMC9220970; doi:10.3390/biom12060841)
Supplement: Supplementary file 1 [file biomolecules-12-00841-s001.zip › SUP2-train_set.pdf]

1a62A	1dyqA	1fp2A	1h2cA	1isuA	1k20A	1lo7A	1n62A
1a7sA	1dysA	1fr3A	1h32A	1it2A	1k38A	1lq9A	1n62B
1a8dA	1dzkA	1fsgC	1h32B	1itxA	1k3iA	1lqtA	1n62C
1abaA	1e29A	1ft5A	1h4aX	1iu8A	1k3xA	1lqvA	1n7oA
1ah7A	1e2wA	1fx2A	1h4xA	1iujA	1k4iA	1ls1A	1n7sA
1ahoA	1e4mM	1fyeA	1h5qA	1iuqA	1k4nA	1lu4A	1n7sB
1aoeA	1e58A	1g12A	1h80A	1ix9A	1k5cA	1lucA	1n7sC
1atgA	1e5kA	1g2qA	1h97B	1ixhA	1k5nA	1lucB	1n7sD
1b0bA	1e6uA	1g2rA	1h99A	1iy8A	1k5nB	1lv7A	1n8vA
1b0uA	1e7lA	1g2yA	1hdhB	1iybA	1k7cA	1lwbA	1na3B
1b5eA	1e9gA	1g3pA	1hdoA	1j0pA	1k7jA	1lzlA	1nbuA
1b67A	1eajA	1g4iA	1hfeM	1j2jB	1k7kA	1m15A	1nc5A
1b6gA	1eaqA	1g5aA	1hfeT	1j2rA	1k8uA	1m1fA	1nc7A
1b9oA	1eazA	1g61A	1hlqA	1j34A	1ka1A	1m1qA	1nfpA
1bgfA	1eb6A	1g66A	1hnjA	1j3aA	1kafA	1m22A	1ng6A
1bkrA	1edmB	1g6gA	1hq1A	1j3wA	1kb0A	1m2dA	1nkdA
1brtA	1egwA	1g6hA	1hqsA	1j77A	1kdgA	1m2xA	1nkgA
1bteA	1ehdA	1g6iA	1ht6A	1j8uA	1kgdA	1m40A	1nkiA
1bx7A	1ej0A	1g6xA	1hw1A	1j98A	1kgsA	1m4iA	1nlqA
1byiA	1ej8A	1g8aA	1hxhA	1jb3A	1kjqA	1m4jA	1nn5A
1c0pA	1ekqA	1ga6A	1hxiA	1jbeA	1kkoA	1m4lA	1nnfA
1c1dA	1elkA	1gciA	1hyoA	1jcdA	1kmtA	1m55A	1nnlA
1c1kA	1eluA	1gheA	1hz4A	1je0A	1kmvA	1m65A	1nnxA
1c4qD	1elwA	1gk7A	1hztA	1jekA	1kngA	1m70A	1nogA
1c52A	1ep0A	1gk9A	1i0rA	1jekB	1knmA	1m9zA	1noxA
1c5eA	1es5A	1gk9B	1i0vA	1jerA	1koeA	1mb3A	1nqjA
1c75A	1es9A	1gkmA	1i12A	1jetA	1kq1A	1mbaA	1nthA
1c7kA	1et1A	1gkpF	1i1nA	1jf3A	1kq3A	1mc2A	1ntvA
1cc8A	1eu1A	1gmuA	1i1wA	1jf8A	1kq6A	1mf7A	1nu0A
1ccwC	1euvA	1gmxA	1i24A	1jfbA	1kqfA	1mg7A	1nuyA
1ccwD	1euwA	1gnlA	1i27A	1jfuA	1kqfB	1mj4A	1nwwA
1cg5A	1evlA	1goiA	1i2tA	1jg1A	1kqfC	1mj5A	1nwzA
1cg5B	1ew0A	1gp0A	1i4uA	1jhjA	1kqpA	1mjnA	1nxcA
1cruA	1ew4A	1gppA	1i52A	1ji7A	1kr4A	1mk0A	1nxmA
1cs1A	1eyvA	1gqiA	1i58A	1jigA	1krhA	1mkkA	1nycA
1cseI	1ezgA	1gqvA	1i60A	1jkeA	1kt6A	1mkzB	1nykA
1cxqA	1f0lA	1gs5A	1i8oA	1jkxA	1kthA	1mn8A	1nz0A
1cy5A	1f1eA	1gtvA	1id0A	1jl0A	1kw3B	1mnnA	1nzjA
1d2sA	1f2tA	1gu2B	1idpA	1jl1A	1kwfA	1moqA	1o04A
1d4oA	1f2tB	1gutA	1ifcA	1jm1A	1kwgA	1ms9A	1o06A
1d4tA	1f46A	1gv9A	1ifrA	1jndA	1kyfA	1mtpA	1o1zA
1d5tA	1f74A	1gvdA	1ig5A	1jniA	1l3kA	1mtpB	1o2dA
1d8wA	1f7dB	1gveB	1ii5A	1jnrA	1l6rA	1munA	1o4yA
1dbwA	1f7lA	1gvpA	1ijqA	1jnrB	1l7aA	1muwA	1o6vA
1dciA	1f86A	1gvzA	1ikpA	1jo0B	1l7mA	1mv8D	1o7iA
1dcsA	1f94A	1gweA	1in4A	1jovA	1l9lA	1mwqB	1o7jA
1dd9A	1f9vA	1gwmA	1inlA	1jr8A	1l9xA	1mxgA	1o8xA
1dfmA	1fcqA	1gxmA	1io0A	1jtvA	1lamA	1mxrA	1o97C
1dg6A	1fcyA	1gxuA	1io7A	1ju2A	1lc0A	1my7A	1o97D
1di6A	1fg7A	1gy7A	1iomA	1juhD	1lc5A	1n08A	1o98A
1dj0A	1fgyA	1gyoA	1iooA	1jx6A	1lf7A	1n13A	1o9gA
1dk8A	1fiuA	1gyxA	1iq6A	1jy2N	1lkkA	1n13B	1o9iA
1dlwA	1fj2A	1gz2A	1iqqA	1jy2O	1llfA	1n3lA	1oaaA
1dp7P	1fm0D	1h12A	1iqzA	1jy2P	1llmC	1n40A	1oaiA
1ds1A	1fm0E	1h16A	1irqA	1jykA	1lmiA	1n45A	1oc7A
1dypA	1fo8A	1h1nB	1is3A	1jz8C	1lniA	1n57A	1ocyA

1od3A	1q4uA	1rk6A	1t6cA	1upqA	1vl7A	1wpuA	1xvxA
1odmA	1q5yA	1rkiA	1t6fA	1ur1A	1vlyA	1wqjB	1xyzA
1odzB	1q6oB	1rkqA	1t6uA	1ursA	1vmgA	1wr8A	1y07A
1oewA	1q6zA	1rkuA	1t8kA	1us0A	1vmhA	1ws8A	1y0hA
1of8B	1q7eA	1rl0A	1t92A	1us5A	1vmjA	1wt6A	1y0pA
1ofwA	1q7lA	1rm6A	1t9hA	1uscA	1vp8A	1wtjA	1y0uA
1oh4A	1q7lB	1rm6B	1t9iA	1useA	1vqsA	1wu4A	1y0yA
1ohlA	1qauA	1rocA	1tbfA	1usmA	1vr7A	1wu9A	1y1pA
1ohpA	1qddA	1rp0A	1tc1A	1utgA	1vrmA	1wvfA	1y2kA
1oi0A	1qe3A	1rtqA	1tg0A	1utiA	1vyiA	1wvhA	1y2mA
1oi6A	1qftA	1rttA	1tgxA	1uuqA	1vykA	1wvqA	1y43A
1oi7A	1qg8A	1ru4A	1thxA	1uuyA	1vyrA	1wwiA	1y43B
1ok0A	1qgvA	1rutX	1tjoA	1uwcB	1vziA	1wy3A	1y4wA
1okiA	1qh5A	1rv9A	1tjxA	1uwkA	1vzmA	1wzaA	1y55X
1olrA	1qhqA	1rw1A	1tkeA	1uz3A	1w0hA	1wzdA	1y5hA
1oohA	1qksA	1rwhA	1tkjA	1uzkA	1w0nA	1x0tA	1y6xA
1oqjA	1qlwA	1ry6A	1tp6A	1v05A	1w0pA	1x1kF	1y8aA
1oqvA	1qnrA	1rylA	1tqgA	1v0wA	1w1hA	1x2iA	1y93A
1orrA	1qowD	1ryoA	1tt8A	1v2xA	1w23A	1x46A	1y9lA
1ou8B	1qq5A	1ryqA	1tu7A	1v30A	1w2lA	1x54A	1y9zA
1ouwA	1qqfA	1s1dA	1tu9A	1v37A	1w4sA	1x6iB	1yb3A
1ow4A	1qs1A	1s1fA	1tuaA	1v3wA	1w53A	1x6oA	1ybkA
1ox0A	1qtnB	1s29A	1tukA	1v4pA	1w5qA	1x6zA	1yc5A
1oxxK	1qtwA	1s2oA	1tvgA	1v5dA	1w5rB	1x7dA	1yd0A
1oygA	1qv1A	1s3cA	1tvnA	1v5iB	1w66A	1x8qA	1yd9A
1oz2A	1qv9A	1s8nA	1tzpA	1v5vA	1w6sA	1x91A	1ye8A
1oznA	1qveA	1s9rA	1tzvA	1v6pA	1w6sB	1x9dA	1yfqA
1p0hA	1qw2A	1s9uA	1u07A	1v70A	1w70A	1x9iA	1yg9A
1p0zA	1qw9B	1sauA	1u0kB	1v7rA	1w7cA	1xbiA	1ylxA
1p1mA	1qwgA	1sbyA	1u2hA	1v7wA	1w9sA	1xd3A	1ymtA
1p1xA	1qwkA	1senA	1u53A	1v7zA	1wb4A	1xdnA	1yn3A
1p3cA	1qwoA	1sfsA	1u69A	1v8cA	1wcuA	1xdzA	1yn9A
1p4cA	1qxyA	1sfxA	1u7gA	1v8hA	1wcwA	1xeoA	1ynpB
1p4oA	1r0mA	1sg4A	1u7iA	1v9yA	1wddA	1xg0A	1ypyA
1p5dX	1r26A	1sh8A	1u84A	1vbwA	1wddS	1xg0C	1yqsA
1p5zB	1r29A	1shuX	1u8vC	1vc3A	1werA	1xg4A	1ys1X
1p6oA	1r2qA	1sj1A	1u9cA	1vccA	1wfbA	1xg5A	1ys7A
1p9gA	1r45A	1sjwA	1uaiA	1vd6A	1whiA	1xgkA	1yt3A
1p9hA	1r55A	1sjyA	1uasA	1vdwA	1whzA	1xjuA	1yu0A
1p9iA	1r5lA	1sk7A	1ucdA	1ve4A	1wkqA	1xk7A	1yuzA
1pb7A	1r5mA	1smoA	1ucrA	1vefA	1wkrA	1xknA	1z0nC
1pbjA	1r62A	1sn9A	1ucsA	1vfyA	1wl8A	1xlqA	1z0wA
1pe9A	1r6dA	1snnA	1uf5A	1vh5A	1wluA	1xmkA	1z1sA
1pkhA	1r6jA	1so7A	1ufoA	1vhnA	1wlyA	1xmtA	1z2nX
1pkoA	1r6xA	1sqsA	1ufyA	1vhtA	1wlzA	1xocA	1z2uA
1pmhX	1r7jA	1svfA	1ug6A	1vhuA	1wm3A	1xodA	1z3eA
1po5A	1r8sE	1svfB	1ugiA	1viaA	1wmaA	1xppA	1z3eB
1pp0A	1r9lA	1svsA	1ugxB	1vimA	1wmdA	1xqoA	1z3xA
1psrA	1ra0A	1sx5A	1uheA	1vioA	1wmhA	1xrkA	1z67A
1pvmA	1rcqA	1sz7A	1ui0A	1vjkA	1wmhB	1xs0A	1z6mA
1pwaA	1rdqI	1szhA	1ujpA	1vjuA	1wmsA	1xszA	1z6nA
1pz4A	1rfyA	1t1gA	1uk8A	1vk1A	1wmwA	1xt5A	1z72A
1pz7A	1rg8B	1t1uA	1ukfA	1vkeA	1wn2A	1xtaA	1zceA
1q0rA	1rgyA	1t1vA	1ukkA	1vkiA	1wnaA	1xteA	1zdyA
1q1fA	1rh9A	1t3yA	1unqA	1vkkA	1wnyA	1xu9A	1zhvA
1q35A	1rjuV	1t61A	1uoyA	1vl1A	1wpaA	1xubA	1zhxA

1zi8A	2b97A	2c78A	2dhoA	2f46A	2g8sA	2hhgA	2ivyA
1zjaA	2b9dA	2c8sA	2dkjA	2f5tX	2gb4B	2hi0A	2iw1A
1zk4A	2bayA	2c92A	2dkoA	2f60K	2gcuA	2hinA	2iwrA
1zk5A	2bbrA	2c9jH	2dkoB	2f62A	2gecA	2hiyA	2ixmA
1zkeA	2bceA	2calA	2dlbA	2f69A	2gf3A	2hlrA	2iyvA
1zkpA	2bcmB	2carA	2dpfA	2f6uA	2ggcA	2hlyA	2izrA
1zl0A	2bdrA	2cb8A	2dplA	2f8aA	2ghsA	2ho2A	2izxA
1zmaA	2bf6A	2cbzA	2driA	2f8yA	2gj3A	2hoxD	2j05A
1zmmA	2bf9A	2cc0A	2drtA	2f9hA	2gj4A	2hp0A	2j1aA
1zoiA	2bfdA	2cc6A	2drxA	2faoA	2gkeA	2hpwA	2j1vA
1zr6A	2bfdB	2ccqA	2ds5A	2fb6A	2gkgA	2hqsH	2j2jA
1zuuA	2bgkA	2ccvA	2dskA	2fbaA	2gkpA	2hqxA	2j43A
1zuyA	2bh4X	2cdcB	2dt4A	2fcjA	2gl5A	2hs1A	2j5gA
1zv1B	2bhuA	2cdpA	2dt8A	2fclA	2glzA	2hsjA	2j5yA
1zx6A	2bjdA	2ce0A	2dtjA	2fcoA	2gmwA	2htdA	2j6bA
1zzkA	2bjiA	2ce2X	2dtxB	2fctA	2gmyA	2huhA	2j6lA
1zzwA	2bjkA	2cf7E	2dvmA	2fcwA	2gomA	2hw2A	2j6vA
2a0bA	2bk9A	2cg7A	2dwuA	2fcwB	2gpiA	2hwnE	2j73B
2a26A	2bkfA	2ci1A	2dxaA	2fdnA	2gqtA	2hx0A	2j82A
2a35A	2bklA	2ciaA	2dxuA	2fe5A	2gqwA	2hx5A	2j8bA
2a3nA	2bkmA	2cibA	2dy0A	2fg1A	2grrB	2hxsA	2j8kA
2a4xA	2bkxA	2cirA	2dy1A	2fgqX	2gs5A	2hykA	2j9oC
2a6zA	2bl8A	2ciuA	2e0qA	2fh1A	2gs8A	2i24N	2j9wA
2a8nA	2blnA	2ciwA	2e3hA	2fhpA	2gsoA	2i33A	2jaeB
2a8yA	2bmoA	2cjtA	2e3nA	2fhzA	2gu9A	2i3dA	2jbaA
2absA	2bmoB	2ckkA	2e4tA	2fhzB	2gudB	2i49A	2jc5A
2agkA	2bo9B	2cksA	2e5fA	2fi1A	2guhA	2i4aA	2jc9A
2ahfA	2bogX	2cm2A	2e6fA	2fj8A	2guiA	2i51A	2jcbA
2aibA	2bpdA	2cnqA	2e7zA	2fkkA	2gwmA	2i53A	2jdaA
2aiqA	2brfA	2covD	2eabA	2fl4A	2gxgA	2i5fA	2jdcA
2akfA	2bryA	2cpgA	2eaqA	2flhA	2gxqA	2i5uA	2je6A
2akzA	2bsyA	2cs7A	2eb4A	2fmaA	2gyqA	2i5vO	2je6B
2amlA	2bt6A	2cuaB	2ebbA	2fnuA	2gz4A	2i61A	2je6I
2anxA	2bt9C	2cveA	2ecuA	2fp1A	2gzqA	2i8tA	2jekA
2ap3A	2bv2A	2cviA	2egvA	2fq3A	2gzsA	2ia1A	2jenA
2apjA	2bw8A	2cwsA	2eh3A	2fr5A	2gzvA	2ia7A	2jepA
2arcA	2bwfB	2cxaA	2ehgA	2frgP	2h1vA	2iayA	2jfrA
2arrA	2bwqA	2cxnA	2ehpA	2fsqA	2h2bA	2ibdA	2jg0A
2asbA	2bwrA	2cxyA	2ehzA	2fsrA	2h30A	2ibnA	2jhfB
2asfA	2bz1A	2cyjA	2eiyA	2ftrA	2h3lA	2ic2A	2jilA
2askA	2bzvA	2cz2A	2ekpA	2fulA	2h5cA	2ic6A	2jisA
2axwA	2c07A	2czlA	2elcA	2fupA	2h6fA	2iciA	2jjnA
2aydA	2c0aA	2czqA	2endA	2fvyA	2h6fB	2icrA	2jjuA
2ayhA	2c0cA	2czsA	2eplX	2fwhA	2h7zB	2ii2A	2jkhL
2b06A	2c1vA	2d1sA	2erfA	2fyfA	2h8eA	2ij2A	2jkuA
2b0aA	2c2nA	2d3dA	2et1A	2g1uA	2h8gA	2ilkA	2jliA
2b0vA	2c2uA	2d3yA	2ev1A	2g2cA	2ha8A	2imfA	2mcmA
2b2hA	2c3nA	2d5mA	2evbA	2g30A	2halA	2imhA	2mhrA
2b3fF	2c3vA	2d5wA	2eveA	2g3rA	2hbaA	2imjA	2nliA
2b3gB	2c46A	2d68A	2ew0A	2g5rA	2hbwA	2imqX	2nlrA
2b4hA	2c4jA	2d8dA	2ex2A	2g6fX	2hc1A	2inwA	2nlsA
2b5aA	2c5aA	2ddrA	2f01A	2g6yA	2hd9A	2ip6A	2nlvA
2b5wA	2c60A	2ddxA	2f1kA	2g7oA	2hdoA	2it2A	2nmlA
2b69A	2c61A	2de3A	2f22A	2g7sA	2heuA	2iu5A	2nn5A
2b7uA	2c6uA	2debA	2f23A	2g81I	2hewF	2iumA	2nnuA
2b82A	2c71A	2dejA	2f3yB	2g84A	2hhcA	2iuwA	2nnuB

2nqtA
2nqwA
2nr7A
2nrrA
2nszA
2nuhA
2nw8A
2nwfA
2nwrA
2nxvA
2nxwA
2o0aA
2o0bA
2o0mA
2o1qA
2o2xA
2o5gB
2o60B
2o6nA
2o6pA
2o6yA
2o7aA
2o7iA
2o7rA
2o8qA
2o90A
2o9sA
2o9uX
2oa2A
2oaaA
2ob3A
2ob5A
2oc3A
2octA
2odiA
2odkA
2ofcA
2ofkA
2ofzA
2og5A
2oh1A
2ohwA
2oizA
2oizD
2okfA
2oktA
2olmA
2olnA
2omlA
2ooaA
2oocA
2opcA
2opgA
2opjA
2oplA
2oqgA
2oqzA
2orwA
2os0A
2os5A
2osxA
2ou5A
2ousA
2ov0A
2ovgA
2ovjA
2oxcA
2oxgY
2oxgZ
2oy9A
2oyoA
2ozhA
2ozjA
2oznA
2oznB
2oztA
2p0nA
2p0sA
2p14A
2p17A
2p2sA
2p39A
2p4fA
2p4hX
2p51A
2p5kA
2p6wA
2p7oA
2p8iA
2p9wA
2pa7A
2pagA
2pbdV
2pc1A
2pefA
2pezA
2pfiA
2pgeA
2pgnA
2phnA
2pieA
2pkfA
2plrA
2plxB
2pm1A
2pn6A
2pndA
2pneA
2pofA
2posA
2pq7A
2pq8A
2pqcA
2pqxA
2pr7A
2prvA
2prxA
2psfA
2pu3A
2pv2A
2pvbA
2pwaA
2pxxA
2py5A
2pyqA
2pyxB
2q0lA
2q1sA
2q2fA
2q3gA
2q3tA
2q3wA
2q52A
2q5cA
2q7dA
2q7wA
2q8kA
2q9kA
2q9oB
2qa9E
2qb7A
2qcpX
2qe8B
2qf4A
2qfaA
2qfaB
2qfaC
2qfeA
2qguA
2qhlA
2qhsA
2qikA
2qipA
2qjlA
2qjwD
2qjzB
2qkvA
2ql8A
2qltA
2qmlA
2qngA
2qnkA
2qnlA
2qntA
2qpxA
2qr6A
2qrlA
2qsbA
2qskA
2qswA
2qt1A
2qudA
2qvgA
2qvkA
2qxfA
2qzcA
2r01A
2r0xA
2r16A
2r1jL
2r2zA
2r31A
2r4iB
2r5oA
2r6jA
2r6uA
2r6vA
2r78A
2r8eA
2r8qA
2r9fA
2ra6A
2ra9A
2rafB
2rb7A
2rb8A
2rbdB
2rbkA
2rc3A
2rc8A
2rdgA
2rdqA
2re2A
2rffA
2rfrA
2rfvA
2rh2A
2rhfA
2rhwA
2rikA
2rilA
2rk3A
2rk5A
2rk9A
2rklA
2rknA
2rkqA
2rkvA
2rl8B
2tpsA
2uu8A
2uuqA
2uuyB
2uv4A
2uvkA
2ux9A
2uxwA
2uy2A
2uytA
2v03A
2v1mA
2v1qB
2v25A
2v27A
2v33A
2v3gA
2v3iA
2v3zA
2v4xA
2v52M
2v6kA
2v6uA
2v6vA
2v76A
2v7fA
2v89A
2v8fA
2v8tB
2v9lA
2v9vA
2vb1A
2vbfA
2vbkA
2vc8A
2vchA
2vclA
2vcnA
2vd8A
2ve8A
2vezA
2vfoA
2vfrA
2vh3A
2vhaA
2vifA
2vimA
2vk2A
2vk8A
2vlaA
2vmhA
2vn6A
2vngA
2vokA
2vovA
2vpaA
2vpnA
2vptA
2vq2A
2vq8A
2vqpA
2vqrA
2vuvA
2vw8A
2vwsA
2vxnA
2vxtI
2vy8A
2vyoA
2vzcA
2vzpA
2w15A
2w1jA
2w1rA
2w1sA
2w1vA
2w31A
2w39A
2w3gA
2w3pA
2w3qA
2w3zA
2w40A
2w50A
2w5aA
2w5qA
2w6aA
2w72D
2w7aA
2w7zA
2w87B
2w8tA
2w8xA
2w91A
2w9hA
2wagA
2wawA
2wb9A
2wbqA
2wcjA
2wcwA
2wdcA
2wdsA
2we5B
2wfiA
2wfwA
2wh6A
2wh7A
2wi8A
2wiyA
2wj5A
2wk1A
2wkjA
2wkkB
2wlrA
2wlvA
2wmfA
2wn3B
2wnfA
2wnkA
2wnpF
2wnvB
2wnvD
2wolA
2woyA
2wq4A
2wqfA
2wqkB
2wraA
2wryA
2wsbA
2wsdA
2wtgA
2wtmA
2wtpA
2wu9B
2wuhA
2wuhB
2wujA
2wvfA
2ww6A
2wweA
2wwxB
2wy4A
2wz8A
2wz9A
2wzbA
2wzoA
2x1fA
2x2sA
2x32A
2x3hB
2x3mA
2x46A
2x49A
2x4kA
2x4lA
2x5nA
2x5oA
2x5pA
2x5xA
2x5yA
2x8sB
2x9gA
2x9oA
2x9zA
2xdpA
2xdwA
2xepA
2xetA
2xeuA
2xevA
2xfdA
2xfrA
2xguA
2xhfB
2xhgA
2xhiA
2xi8A
2xioA
2xirA
2xj4A
2xjpA
2xkiA
2xmjA
2xn6A
2xn6B
2xnqA
2xodA
2xolA
2xomA
2xpwA
2xqqA
2xrhA
2xryA
2xs2A
2xtpA
2xtsA
2xtsB
2xttA
2xu3A
2xuvA
2xvmA
2xw6A
2xw9A
2xwlA
2xwsA
2xwvA
2xz2A
2xziB
2y0oA
2y27A
2y3cA
2y53A
2y5pD
2y6xA
2y71A
2y78A
2y7eA
2y8kA
2y8yA
2y9uA
2ybyA
2yc3A
2ycdA
2yd6A
2yexA
2yfoA
2yh5A

2yh6A	2zw2A	3ayjA	3bqxA	3covA	3ds4A	3exvA	3fynA
2yhgA	2zwsA	3azdA	3br8A	3cp5A	3dsbA	3ey6A	3fz4A
2yimA	2zxyA	3b0fB	3brcA	3cp7A	3dskA	3eyeA	3g0kA
2ykzA	2zyoA	3b0gA	3bs4A	3crnA	3dsoA	3eyiA	3g16A
2ylnA	2zzvA	3b0sA	3bt5A	3ct5A	3duwA	3f0dA	3g1pA
2ymvA	3a02A	3b0tA	3buuA	3ct6A	3dwgA	3f14A	3g21A
2yn0A	3a07A	3b0xA	3buxB	3ctpA	3dwgC	3f1lA	3g36C
2yogA	3a09A	3b1nA	3bvfA	3ctzA	3dxlA	3f1pB	3g46A
2yv9A	3a0mA	3b34A	3bvxA	3cu9A	3dxyA	3f2zA	3g48A
2yveA	3a0sA	3b4nA	3bwhA	3cuzA	3e0eA	3f40A	3g4eA
2yvtA	3a0yA	3b4qA	3bwvB	3cvbA	3e0xA	3f43A	3g5sA
2ywiA	3a16A	3b4uA	3bwxA	3cwnA	3e10B	3f44A	3g5tA
2ywkA	3a1hA	3b5mA	3bwzA	3cwrB	3e23A	3f4sA	3g7nA
2ywlA	3a1sA	3b5nA	3by4A	3cx2A	3e2dA	3f5vA	3g7rA
2yxmA	3a2zA	3b5nC	3by8A	3cxnA	3e2oA	3f6cA	3g89A
2yxoA	3a35A	3b5nD	3c2uA	3cypB	3e2vA	3f6vA	3g91A
2yykA	3a4rA	3b5oA	3c3yA	3cz1A	3e3mA	3f6yA	3g9yA
2yzvA	3a57A	3b64A	3c5eA	3czxA	3e3uA	3f7eA	3ga3A
2yzyA	3a5fA	3b79A	3c6aA	3d02A	3e48A	3f7xA	3ga4A
2z08A	3a6rA	3b9tB	3c70A	3d06A	3e4gA	3f8dA	3ga7A
2z0jA	3a72A	3b9wA	3c7mA	3d0jA	3e4vA	3f8xD	3gaeA
2z26A	3a8gA	3ba1A	3c8cA	3d1pA	3e7rL	3f9mA	3gbwA
2z3hA	3a8gB	3ba3A	3c8lB	3d22A	3e8mD	3f9xA	3gd6A
2z4uA	3a99A	3bb0A	3c8wC	3d2qA	3e8oB	3fbgA	3ge3A
2z51A	3a9fA	3bb7A	3c8zA	3d3bA	3e8tA	3fcnA	3ge3B
2z5wA	3a9jC	3bc9A	3c9aA	3d3bJ	3e8yX	3fdeA	3ge3C
2z6oA	3a9sA	3bcwB	3c9uA	3d40A	3e9tA	3fdxA	3ge3E
2z6rA	3a9zA	3bd1A	3c9zA	3d4eA	3ea6A	3fedA	3gfaA
2z72A	3aalA	3bdiA	3ca7A	3d59A	3eazA	3fegA	3gg7A
2z8xA	3aamA	3bedA	3caoA	3d5pA	3ebtA	3fg9A	3ghaA
2z98A	3abnA	3beuA	3cayA	3d7jA	3ebvA	3fghA	3ghjA
2zadA	3achA	3bexA	3cb0A	3d9aL	3ec6A	3fgvA	3girA
2zatA	3acxA	3bf7A	3cbwA	3d9nA	3ec9A	3fgyA	3giuA
2zblA	3admA	3bfoA	3cbzA	3d9xA	3edhA	3fh1A	3giwA
2zbxA	3agnA	3bguA	3ccdA	3da8B	3ednA	3fiaA	3gj0A
2zcmA	3agtA	3bhdA	3ccgA	3daqA	3edoB	3filA	3gjuA
2zcwA	3ah9A	3bhqB	3cecA	3dasA	3ef8A	3fiqA	3gjyA
2zdpA	3aiaA	3bhwA	3ch0A	3db7A	3einA	3fjuB	3gkjA
2zexB	3aj3A	3bjeB	3chjA	3deoA	3ej9A	3fk8A	3gkrA
2zfdA	3aj4A	3bkrA	3chmA	3dfgA	3ej9B	3fkeA	3gmgA
2zfdB	3aj6B	3bkwB	3chvA	3dg6A	3ejfA	3fmyA	3gmxB
2zfiA	3aj7A	3blnA	3ci3A	3dhaA	3ejvA	3fo3A	3gneA
2zhjA	3ajdA	3bm7A	3ci6A	3di4B	3ekgA	3fojA	3gnlA
2zhnA	3ajxA	3bmvA	3cijA	3djeB	3ekiA	3fpcA	3gnzP
2zk9X	3ak2A	3bmxA	3cimA	3dk9A	3elfA	3fpwA	3go5A
2zl7A	3ak8A	3bmzA	3cjmA	3dkmA	3en0A	3fr7A	3gocB
2znrA	3akbA	3bnjA	3cjsA	3dkrA	3eo6B	3frhA	3goeA
2zouA	3aksA	3bo5A	3cjsB	3dlcA	3eoiA	3fsaA	3gohA
2zpmA	3alfA	3bo6A	3cjwA	3dmgA	3eojA	3fsoA	3goxA
2zptX	3aljA	3boeA	3ckcA	3dnjA	3eqxB	3fssA	3gp2B
2zq0A	3amnB	3bomA	3ckkA	3do8A	3er7A	3ftdA	3gp6A
2zs0A	3amrA	3bonA	3ckmA	3douA	3erpA	3futA	3gpiA
2zs0B	3aofB	3bpkA	3cl6A	3dqpA	3essA	3fw9A	3gpkA
2zs0C	3aowA	3bptA	3clmA	3dqyA	3etjA	3fwkA	3gr3A
2zs0D	3arxA	3bpvA	3cm3A	3dr4A	3eurA	3fxaA	3grdB
2zuxB	3aslA	3bqpA	3cmbD	3drfA	3ewyA	3fymA	3gruA

3gv3A
3gveA
3gwaA
3gwiA
3gwkC
3gxhA
3gy9A
3gybA
3gzaB
3gzbF
3gzrB
3gzxA
3gzxB
3h0nA
3h0oA
3h0uA
3h31A
3h3hA
3h3lB
3h4nA
3h4oA
3h4tA
3h4xA
3h5jA
3h6jA
3h74A
3h75A
3h79A
3h7hA
3h7hB
3h7iA
3h87A
3h87C
3h8gF
3h9mA
3hcnA
3hdxA
3hf5A
3hfoA
3hhpA
3hhtB
3hhyA
3hj4A
3hkwA
3hlxA
3hlzA
3hm4B
3hmcA
3hmzA
3hnxA
3hnyM
3ho6A
3ho7A
3hoiA
3hp7A
3hpcX
3hpwC
3hr6A
3hs3A
3ht1A
3htnA
3hu5A
3huhA
3hupA
3hv2A
3hv8A
3hwuA
3hx8B
3hynA
3hz8A
3hzaA
3hzpA
3i10A
3i2kA
3i2vA
3i2zB
3i3fA
3i45A
3i47A
3i4gA
3i4oA
3i6cA
3i7mA
3i94A
3iarA
3ib5A
3ib7A
3ie4A
3ie7A
3iezA
3ifeA
3igsB
3ihsA
3iisM
3ijlA
3ikwA
3im9A
3imkA
3ip0A
3ipcA
3ipjA
3ipnA
3iqtA
3iquA
3ir4A
3irpX
3irvA
3is3A
3isxA
3it3A
3itfA
3itqA
3iuoA
3iuwB
3iv0A
3iv4A
3ivvA
3ivyA
3ivzA
3iwfA
3iwlA
3ix3A
3ixlA
3jq0A
3jq1A
3jrvA
3js8A
3jsyA
3jtzA
3ju4A
3judA
3jumA
3jxoA
3jyoA
3jyzA
3jzyA
3k01A
3k05A
3k0bA
3k12A
3k1uA
3k1wA
3k1zA
3k21A
3k2zA
3k5jA
3k67A
3k6iA
3k6mA
3k6yA
3k7pA
3kanB
3kb5A
3kb9A
3kc2A
3ke7B
3keoA
3kevA
3kffA
3kgkA
3kgyB
3kh1B
3khfA
3kizA
3kkfA
3kkgA
3km5A
3kmaA
3kmhA
3knbA
3knbB
3korA
3kpbA
3kpeB
3kruA
3ktcB
3ktpB
3ktzB
3ku3B
3kuvA
3kweA
3kwrA
3kxqA
3kyjA
3kyjB
3kyzA
3kz5E
3kzdA
3l12A
3l1nA
3l1wA
3l32A
3l41A
3l46A
3l4aA
3l4eA
3l4nA
3l4rA
3l51A
3l51B
3l5lA
3l77A
3l81A
3l84A
3l8aA
3l8wA
3l9aX
3l9cA
3laaA
3laeA
3lagA
3laxA
3lb2A
3ld7A
3ldcA
3ledA
3lf5A
3lfjA
3lfkA
3lfrA
3lftA
3lg3A
3lhcA
3lhiA
3lhnA
3lhqA
3lhsA
3ljmA
3lk7A
3lkmA
3lloA
3lluA
3lm3A
3lmzA
3lnyA
3lo8A
3lopA
3lq0A
3lqbA
3lqwA
3lrtA
3ls9A
3lsnA
3lt7A
3ltiA
3lw3A
3lwcA
3lwxA
3lx3A
3ly0A
3lydA
3lyeA
3lyhB
3lypA
3m07A
3m0fA
3m0mA
3m0zA
3m3pA
3m5qA
3m66A
3m6wA
3m6zA
3m73A
3m7aB
3m8jA
3m9lA
3m9qA
3mabA
3maoA
3mb5A
3mbkA
3mbrX
3mc3A
3mcwB
3mcxA
3md1A
3md7A
3md9A
3mdmA
3mdqA
3mduA
3me7A
3meaA
3milA
3mjeA
3mjfA
3mk1A
3ml1A
3ml1B
3mmhA
3mn5S
3moyA
3mozA
3mpcA
3mqdA
3mqhA
3mqzA
3mr0A
3mstA
3mt0A
3mu7A
3mvcA
3mvgA
3mvsA
3mwxA
3mwzA
3mxnA
3mxnB
3mxzA
3mybA
3myxA
3mz2A
3mzfA
3n01A
3n08A
3n0rA
3n0uA
3n0xA
3n10A
3n17A
3n1fC
3n2wA
3n3mA
3n4iB
3n4jA
3n6yA
3n6zA
3n79A
3nbcA
3nbmA
3nd1A
3ndhA
3ndoA
3ne8A
3nedA
3nepX
3neqA
3neuA
3nfkA
3nftA
3nfwA
3nggA
3ngpA
3nhiA
3nirA
3nj2A
3njnA
3nkeA
3nnbA
3no0A
3no2A
3no7A
3nohA
3noqA
3npdA
3npkA
3nr5A
3nreD
3nrfB
3ns6A
3ntvA
3nuaA
3nufA
3nvsA
3nvwA
3nvwB
3nvwC
3nwpA
3nwrA
3nycA
3nyqA
3nytA
3nyyA
3nzbX
3nzlA
3nznA
3o12A
3o22A
3o2rA
3o2tA
3o4pA
3o7bA
3o8mA
3o8qA
3o94A
3o9zA
3oa3A
3oajA
3oblA
3obqA
3oc7A
3ocjA
3ocuA
3odtB
3odvA
3oe3A
3of5A
3ofgA
3og2A
3ognA
3oheA
3om0A
3omdA
3omyA
3on9A
3ondA
3oo8A
3oooA
3oouA
3ooxA
3oqpA
3orkA
3oruA
3otiA
3ou2A
3ov5A
3ov9A
3ox7P
3oxpA
3oyvA
3oz2A
3ozyA
3p02A
3p0bA
3p0fA
3p0kA
3p1gA
3p3oA
3p4hA
3p8gA
3pa6A
3pb6X
3pd7A
3pe6A
3pe8A
3pesA
3peyA
3pf6A
3pfbA
3pfeA
3pfgA
3phhA
3pidA
3piuA
3piwA
3pjpA
3pjyA

3pkvA
3pl8A
3pluA
3plwA
3pmcA
3pmeA
3pmoA
3pmsA
3pn3B
3pnaA
3pnzA
3po8A
3podA
3pojA
3ponA
3powA
3pp2A
3pp4P
3pp5A
3pp9A
3pplA
3pqaA
3pqhA
3psmA
3pt5A
3pu9A
3pucA
3pveA
3pvhA
3pviA
3px8X
3pzgA
3q1cA
3q1xA
3q2bA
3q2iA
3q46A
3q4oA
3q64A
3q6bA
3q7rA
3q8jA
3qatA
3qb8A
3qc0A
3qc7A
3qftA
3qguB
3qhbA
3qhpA
3qjaA
3qk8A
3ql9A
3qnsA
3qooA
3qp4A
3qpaA
3qr7A
3qsdA
3qu3A
3qu5A
3qvpA
3qwbA
3qx1A
3qxcA
3qxzA
3qy1A
3qzbA
3qzmA
3qzrA
3qzxA
3r0nA
3r0vA
3r2qA
3r3qA
3r3rA
3r3sA
3r41A
3r4zA
3r5gA
3r5tA
3r62A
3r68A
3r6dA
3r72A
3r87A
3r8jA
3r9fA
3rd5A
3rfeB
3rgaA
3rhbA
3rhgA
3riqA
3rjtA
3rjuA
3rkgA
3rl5A
3rlgA
3rm3A
3rnjA
3rnqB
3ro3A
3ro8A
3robA
3rofA
3rpcA
3rpdA
3rpeA
3rpzA
3rq9A
3rqtA
3rr6A
3rriA
3rt2A
3rtlA
3rwnA
3rx9A
3ry0A
3ry4A
3rznA
3s0aA
3s2jA
3s2rA
3s44A
3s4eA
3s57A
3s5mA
3s6eA
3s6fA
3s83A
3s8mA
3s8sA
3s9xA
3sbmA
3sc7X
3scyA
3sd2A
3sebA
3seeA
3sfjA
3sg0A
3sggA
3sh4A
3shgA
3shgB
3sigA
3sjmA
3sk2A
3sk7A
3skxA
3snfA
3snoA
3so6A
3soeA
3sojA
3sovA
3sp7A
3sqzA
3sriB
3ss7X
3su6A
3sukA
3suuA
3swoA
3sx2A
3sxmA
3sxxA
3sy1A
3sz3A
3szaA
3szhA
3szvA
3szyA
3t0oA
3t2cA
3t3lA
3t47A
3t49A
3t4lA
3t7hA
3t7lA
3t7vA
3t8jA
3t90A
3t92A
3t9wA
3ta6A
3tbnA
3tboA
3tc2A
3tc3A
3tc8A
3td3A
3tdnA
3tduD
3teuA
3tewA
3tfjA
3tg0A
3tg2A
3tg7A
3tj4A
3tj8A
3tjmA
3tjrA
3tm8A
3tn4A
3tnlA
3tnyA
3tosA
3towA
3tpdA
3tq2A
3tq5A
3tqeA
3tqlA
3trdA
3ts3A
3tt9A
3tutA
3tvjA
3tvjB
3tvtA
3tvtB
3ty4A
3tysA
3u2uA
3u3gD
3u3lC
3u3zA
3u5sA
3u62A
3u65B
3u6gA
3u7qA
3u7qB
3u7rA
3u7zA
3u97A
3u99A
3u9rB
3u9wA
3ub6A
3ubdA
3uc7A
3ue2A
3uf7A
3ufeA
3uffA
3ui4A
3uidA
3ujcA
3ujiP
3uk0A
3uljA
3ultA
3unvA
3up3A
3uplA
3upvA
3ur8A
3urrA
3uueA
3uv9A
3uw3A
3uxjA
3v0dA
3v1aA
3v30A
3v46A
3v4gA
3v4kA
3v5cA
3v68A
3v75A
3v7nA
3v7qA
3v9oA
3va4A
3vc5A
3vcaA
3vcxA
3ve9A
3vejA
3venA
3vg7A
3vgiA
3vglA
3vi6A
3viiA
3vj9A
3vk5A
3vl1A
3vl9A
3vlaA
3vmkA
3vmnA
3vmvA
3vn0A
3vn3A
3vorA
3vqjA
3vrcA
3vrdA
3vrdB
3vsvB
3vtoA
3vubA
3vupA
3vurA
3vv1B
3vviA
3vvvA
3vwcA
3vwnX
3vypA
3vz9B
3vz9D
3vzxA
3w06A
3w07A
3w0kA
3w0oA
3w0tA
3w19C
3w19D
3w42A
3w56A
3w5sA
3w7tA
3w7yA
3w7yB
3wa2X
3wasB
3wdcA
3wdnA
3wdqA
3weoA
3wg3B
3wgxA
3wh1A
3wiwA
3wj1A
3wjpA
3wjtA
3wkgA
3wkqA
3wliA
3wmqA
3wmtB
3wmvB
3wn7B
3wn8A
3wndA
3wp4A
3wpcB
3wqbA
3wqbB
3wqcA
3ws7A
3wucA
3wupA
3wurA
3wuzA
3wv7A
3wvaA
3wvsA
3wwcA
3wwlA
3wwxA
3wx7A
3wy2A
3wydA
3wyeA
3wz3A
3x0iA
3x0tA
3x2mA
3x34A
3zbdA
3zboB
3zdbA
3zfpA
3zhiA
3zhnA
3zhoA
3zitA
3ziyA
3zjaA
3zmrB
3zn4A
3zn6A
3znvA
3zojA
3zoqB
3zpyA
3zqxA
3zr8X
3zrxA
3zsjA
3zsuA
3ztvA
3zucA
3zuzA
3zvsA
3zxcA
3zxfA
3zxkA
3zxyA
3zy2A
3zy7A
3zypA
3zyqA
3zzoA
3zzpA
3zzsA
3zzyA
4a02A
4a14A
4a29A
4a2vA
4a37A
4a3pA
4a3zA
4a42A
4a4jA
4a4yA
4a56A
4a6hA
4a6qA
4a6rA
4a7uA
4a8tA
4a9vA
4aaaA
4aazA
4ac1X
4acjA
4ae7A
4af8A
4affA
4afmA
4ag1A
4ag7A
4agiA
4ak2A
4ak8A

4al0A	4bpfA	4cz5A	4e69A	4f0bA	4gekA	4he6A	4icvA
4alzA	4bpsA	4czgA	4e6fA	4f0jA	4ggcA	4hfqA	4id0A
4ammA	4bpyA	4d0pA	4e6uA	4f0wA	4ggfA	4hfsA	4id9A
4annA	4bpzA	4d0qA	4e74A	4f1vA	4ggfC	4hg2A	4idcA
4ao6A	4bqnA	4d6gA	4e9oX	4f2eA	4ghnA	4hguA	4iedA
4ao9A	4brcA	4d74A	4e9sA	4f2fA	4gieA	4hhrA	4iejA
4aohA	4bt7A	4d7jA	4e9xC	4f2lA	4gjzA	4hi7A	4ifaA
4aqoA	4bu0A	4d8bA	4ea9A	4f3jA	4gmqA	4hi8A	4igiA
4aruA	4bvxA	4db5A	4eadA	4f54A	4gmuA	4hi8B	4iheA
4at0A	4bvxB	4dd5A	4eaeA	4f66B	4gneA	4hjbC	4ihmA
4ateA	4by8A	4df0A	4ebgA	4f87A	4gnrA	4hjiA	4ihzA
4au1A	4byzA	4dgfA	4ebjA	4f8xA	4gofA	4hkgA	4iikA
4avrA	4bz4A	4di9A	4ecfA	4f98A	4gosA	4hlyA	4iilA
4avsE	4c08A	4djaA	4ee6A	4fayA	4gqmA	4hmsA	4iiyA
4aw7A	4c1aA	4dk2A	4ee9A	4fb2A	4grzA	4hnlA	4ij5A
4aweA	4c24A	4dm5A	4eekA	4fbjA	4gs3A	4hnoA	4ikdA
4axoA	4c2vA	4dm7A	4eewA	4fchB	4gt8A	4hojA	4il7A
4axyA	4c2vC	4dmiA	4ef0A	4fflA	4gt9A	4hpnA	4in0A
4ay0A	4c5kA	4dmtA	4efiA	4fglA	4gucA	4hq1A	4in9A
4ayoA	4c6aA	4dmvA	4efpA	4fh0A	4gvfB	4hqzA	4incA
4az6A	4c72A	4dn2A	4eguA	4fk9A	4gvoA	4hroA	4ineA
4b0hA	4c81A	4dn7A	4ehuA	4fn7A	4gvqA	4hs1A	4inkA
4b0zA	4ca1A	4dndA	4eicA	4fnvA	4gvxD	4hs2A	4inwA
4b15A	4cayC	4dnxB	4eihA	4fojA	4gwbA	4hstA	4io2A
4b1mB	4cbpA	4do4A	4eirB	4fp5D	4gwgA	4hstB	4ipuA
4b1yM	4cc2A	4dpbX	4eisA	4fr9A	4gxwA	4htfA	4irxA
4b21A	4cd5A	4dpzX	4eivA	4fs7A	4gymA	4htgA	4itbA
4b4uA	4cd8A	4dq6A	4ekfA	4ftfA	4gyxA	4hvkA	4itcA
4b5oA	4cdjA	4dq9A	4emnA	4fzlA	4gzcA	4hvyA	4iumA
4b62A	4cdpA	4dqaA	4eo7A	4fzpA	4h14A	4hwmA	4iusA
4b6gA	4ce8C	4dqjB	4ep4A	4g0xA	4h15A	4hwvA	4ix3A
4b89A	4cfiA	4dr8A	4eq9A	4g1qA	4h17A	4hy4A	4ix7A
4b8xA	4cfqQ	4driB	4eqaC	4g2eA	4h27A	4hz2A	4iyaA
4b9gA	4cgsA	4dt4A	4eqbA	4g3bA	4h3uA	4hzrA	4iyjA
4b9iA	4chiA	4dt5A	4eqpA	4g3nA	4h4dA	4i0wA	4izbA
4b9pA	4ci7A	4duiA	4eqsA	4g3oA	4h4nA	4i0wB	4izxA
4bb9A	4ci9A	4dvcA	4ercA	4g41A	4h5iA	4i1fA	4j0dA
4bboA	4cicA	4dwdA	4errA	4g4kA	4h6cI	4i1kA	4j0eA
4begA	4cilA	4dwoA	4es1A	4g54A	4h6qA	4i2uA	4j1oA
4beuA	4cj0A	4dwrA	4es8A	4g6tA	4h7pA	4i3gB	4j27A
4bfhA	4cj0B	4dxkA	4esmA	4g6tB	4h7uA	4i4eA	4j3vA
4bfoA	4ck4A	4dyqA	4esrA	4g78A	4h7wA	4i4oA	4j42A
4bgbA	4cndA	4dzhA	4eswA	4g7xA	4h87A	4i62A	4j4zA
4bgcA	4cngA	4dziA	4etnA	4g7xB	4h89A	4i66A	4j5rA
4bguA	4cnnA	4e15A	4eu9A	4g9eA	4h8eA	4i6rA	4j6oA
4bh5A	4co8A	4e19A	4eunA	4g9sA	4ha4A	4i6xA	4j73A
4bj0A	4cogA	4e29A	4euoA	4g9sB	4hbqA	4i6yA	4j7aA
4bjiA	4cp6A	4e2bA	4evqA	4ga2A	4hbzA	4i71A	4j7nA
4bjzA	4cs4A	4e2uA	4evuA	4gb5A	4hc9A	4i7wA	4j7qA
4bk7A	4csrA	4e2xA	4ex6A	4gb7A	4hcjA	4i84A	4j8cA
4bmhA	4csrB	4e3xA	4exkA	4gbuA	4hcsA	4i8hA	4j8sA
4bmnA	4cuaA	4e3yA	4eysA	4gc3A	4hddA	4i8iA	4j9tA
4bn4A	4cv7A	4e40A	4eyzA	4gciA	4hdeA	4i93A	4j9yB
4bndA	4cvrA	4e4rA	4ezgA	4gcoA	4hdrA	4iauA	4jb3A
4boqA	4cw4A	4e4tA	4eziA	4ge6A	4hdrB	4ic4A	4jb7A
4bouA	4cxpA	4e4uA	4f06A	4geiA	4hdtA	4iciA	4jbbA

4jbdA
4jccA
4jduA
4je1A
4jedA
4jejA
4jemA
4jerA
4jf1A
4jf8A
4jg2A
4jgiA
4jglA
4jguB
4jhtA
4jiuA
4jjaA
4jk8A
4jm1A
4jmpA
4jn7A
4jnuA
4jokA
4jp6A
4jqfA
4jtmA
4jvuA
4jwxA
4jxrA
4jz5A
4jzzA
4k0nA
4k12A
4k12B
4k3lA
4k7bA
4k7zA
4k82A
4k8gA
4kalA
4kbxA
4kdwA
4kdxA
4kefA
4kemA
4kg7A
4kgdA
4kh7A
4kh8A
4kl0A
4kliA
4km6A
4kmgA
4kmrA
4kn8A
4knkA
4kqdA
4kqiA
4kqpA
4kruA
4ks7A
4kt3A
4kt3B
4ku0A
4ku0D
4kv7A
4kxvA
4kzvB
4l05A
4l2hA
4l2iA
4l2iB
4l4eA
4l57A
4l5eA
4l6dA
4l7xA
4l8aA
4l8pA
4l9oA
4l9pA
4l9pB
4la2A
4lc3A
4ld1A
4ldcA
4ldvA
4lebA
4lerA
4lf0A
4lgjA
4lgtD
4lgyA
4lhsA
4lixA
4lizA
4ljiB
4lksA
4lkuA
4lldA
4lldB
4lmyA
4lowA
4lplA
4lpqA
4lr2A
4lrtA
4lrtB
4lruA
4lttA
4luaA
4lukA
4lupA
4lvuA
4lwlA
4lx2A
4lx3A
4lx3B
4lxqA
4lypA
4lzxB
4m02A
4m0wA
4m1gA
4m1qA
4m1uA
4m1xA
4m2mA
4m51A
4m5eA
4m5rA
4m7tA
4m82A
4m8aA
4m91A
4m9kA
4m9vF
4maiA
4makA
4mamA
4maqA
4maxA
4mc3A
4mcoA
4me2A
4mf5A
4mfiA
4mijA
4miyA
4mjdA
4mjeA
4mkxA
4mllA
4mlvA
4mmgA
4mncA
4mnoA
4mq3A
4mqbA
4mtmA
4mtuA
4mupA
4muqA
4muvA
4muzA
4mx6A
4mxtA
4mydA
4mykA
4myzA
4mzcA
4mzdA
4mzjA
4n02A
4n03A
4n13A
4n1iA
4n2kA
4n2pD
4n30A
4n3tA
4n4uA
4n5mA
4n5uA
4n67A
4n6kA
4n7fA
4n8cX
4n8gA
4nazA
4nbpA
4nbrA
4nbuA
4ndoA
4ndoB
4ndsA
4necA
4nesA
4nf1A
4nfnA
4ng0A
4ni6A
4nkpA
4nl9A
4nl9C
4nlmA
4nmuA
4nmwA
4nn2A
4nn3A
4nnoA
4noaA
4nobA
4nogA
4nohA
4novA
4npdA
4nsmA
4nsvA
4ntdA
4ntkA
4nutA
4nutB
4nx1B
4nxyA
4nyhA
4nyqA
4nzkA
4o06A
4o0aA
4o0cA
4o0kA
4o5fA
4o6uA
4o7hA
4oa3A
4oanA
4ocvA
4od6A
4odkA
4oe9A
4oelA
4ofaA
4ogdB
4oggA
4oh7A
4ohjA
4ohnA
4oi3A
4ojxA
4okiA
4oltB
4om8A
4ombA
4onmA
4onrA
4opcA
4oq9A
4oqpA
4oqvA
4ou0A
4oujB
4ousA
4oxxA
4oy3A
4oy5A
4oy7A
4p0tA
4p0zA
4p32B
4p3hA
4p3vA
4p40A
4p47A
4p5eA
4p5nA
4p5pA
4p7oA
4p7xA
4p82A
4p8bA
4p9iA
4pakA
4pbhA
4pc9A
4pcaB
4pdnA
4pdyA
4pe0A
4pe3A
4pf3A
4pf4A
4pf8A
4pfyB
4ph2A
4ph8A
4phjA
4phrA
4pi8A
4pioA
4pitD
4pj2A
4pj2C
4pklA
4plzA
4pmoA
4pmxA
4pneB
4pnoA
4powA
4pp4A
4pq9A
4pqdA
4pqhA
4pqqA
4ps6A
4pscA
4psfA
4psrB
4pssA
4puxA
4pvaA
4pvkA
4pw0A
4pwoA
4pxeA
4pxyA
4pyrA
4pz0A
4pz3A
4pzjA
4q27A
4q29A
4q2lA
4q2qA
4q2sA
4q34A
4q3kA
4q4gX
4q53A
4q68A
4q6jA
4q6tA
4q7eA
4q7oA
4q7qA
4q98A
4q9bA
4qa8A
4qa9A
4qasA
4qb3A
4qb6A
4qboA
4qc6A
4qdjA
4qekA
4qf3A
4qgoA
4qhqA
4qhwA
4qi3B
4qi8A
4qitA
4qkdA
4qlpA
4qlpB
4qm6A
4qnsA
4qosA
4qp5A
4qpnA
4qptA
4qpwA
4qqhA
4qqsA
4qrnA
4qt3A
4qtcA
4qucA
4qusA
4qwoA
4qxbB
4qxbC
4qxlA
4qy7A
4qytA
4r03A
4r16A
4r1jA
4r1sA
4r1vA
4r2xC
4r38A
4r3fA
4r3nA
4r52A
4r5rA
4r6hA
4r6rE
4r6yA
4r75A
4r78A
4r81A
4r8hA
4r8xA
4r9fA
4r9pA
4raxA
4rayA
4rbxA
4rcjA
4rd4A
4rd7A
4rdbA
4reiA
4rekA
4reoA
4rexA
4rfuA
4rgdA
4rgyA
4ri5A
4ri6A
4rj2A
4rjwA
4rjzA
4rk4A
4rkfA
4rl3A
4rlcA
4rleA
4rlzA
4rp3A
4rpmA
4rptA
4rqrA
4rriA
4rs2A
4rt5A
4rthA
4ru1A
4ru3A
4ru5A
4ruqB
4ruwA
4rv5A
4rvqA

4rwcA	4udxX	4w8pB	4x1zB	4y2mA	4z0gA	4zvcA	5aogA
4rwuA	4ue0A	4w9zA	4x2rA	4y6wA	4z0tA	4zvfA	5aotA
4rxtA	4ue8A	4wbjA	4x33A	4y7lA	4z0yA	4zw9A	5aovA
4rxuA	4ue8B	4wbtC	4x33B	4y88A	4z1rA	4zwvA	5aozA
4rxvA	4ufqA	4wbyA	4x54A	4y9iA	4z2oA	4zx2A	5apgA
4ry1A	4uhcA	4wcgA	4x5pA	4y9mA	4z39A	4zy9B	5aq0A
4ry9A	4uhoA	4wckA	4x7gA	4y9vA	4z3gA	4zz1A	5aulA
4ryaA	4uhtA	4wcxA	4x84A	4y9wA	4z47A	5a0dA	5avdA
4ryoA	4uiqA	4wdcA	4x8eA	4yaaA	4z4dA	5a0lB	5awoA
4rz9A	4uj7A	4we2A	4x9cA	4yagA	4z4jA	5a0nA	5ax0A
4s12A	4ulvA	4wecA	4x9jA	4yapA	4z67A	5a0yA	5azbA
4s1hA	4umiA	4weeA	4x9rA	4ybmA	4z6mB	5a0yB	5azwA
4s1pA	4un2B	4wepB	4x9tA	4ycbA	4z7eA	5a0yC	5azxB
4s28A	4unuA	4wesB	4x9xA	4ydrB	4z7xA	5a10A	5b08A
4s2xA	4uobA	4wf5A	4x9zA	4ye7A	4z80B	5a12A	5b1aD
4s36A	4up0A	4wfoA	4xa7A	4yecA	4z80C	5a1iA	5b1aE
4s39A	4up3A	4wfvA	4xbaB	4yecB	4z9hA	5a1qA	5b1aG
4s3jA	4upiA	4wh9A	4xcbA	4yepA	4za2D	5a35A	5b1aI
4tkcA	4uqwA	4whsC	4xcvA	4yfuA	4zavA	5a3aA	5b1aJ
4tm7A	4uqxA	4whsD	4xduA	4yg0A	4zbdA	5a57A	5b1aK
4tmxA	4uqzB	4wilA	4xdxA	4ygbD	4zbgA	5a61A	5b1aM
4tpnA	4urfA	4wiqA	4xedA	4yi8A	4zbhA	5a62A	5b1aN
4tpvA	4usaA	4wjiA	4xemA	4yjrA	4zblA	5a67A	5b1aP
4tqrA	4utuA	4wjtA	4xezA	4ykiA	4zboD	5a6mA	5b1aS
4tqxA	4uu3B	4wk7A	4xfjA	4yl8A	4zc3A	5a71A	5b1aU
4tr6A	4uu5A	4wkaA	4xfkA	4yl8B	4zceB	5a7gA	5b1aY
4troA	4uulB	4wlhA	4xfmA	4ylaA	4zd6F	5a7vA	5b1rA
4tsdB	4uwwA	4wn5A	4xfwA	4ymeA	4zeyA	5a8cA	5b1sA
4ttnA	4uybA	4wndB	4xhvA	4ymyA	4zfvB	5a8jA	5b4bB
4ttwA	4uypA	4wnoA	4xhyA	4ynhA	4zgfA	5a95B	5b4tA
4tvvA	4uyrA	4wp4A	4xijA	4ynuA	4zgwA	5a99A	5b4zA
4txrA	4uzgA	4wp9A	4xinA	4ynxA	4zh5B	5a9tA	5b5iA
4txrB	4v0kA	4wpgA	4xj5A	4yorA	4zhbA	5ab8A	5b5lA
4txrC	4v0wB	4wpkA	4xjwA	4ypoA	4zhwA	5achA	5b6cA
4txwA	4v12A	4wpyA	4xlzB	4yqdA	4zilA	5acsB	5b78B
4tyzA	4v15A	4wqdA	4xmhA	4yqyA	4zjhA	5ad1A	5b7gA
4tz1A	4v1gA	4wqkA	4xmrA	4ysiA	4zjuA	5ae0A	5b7hA
4tzhA	4v1jA	4wriA	4xosA	4yslA	4zl8A	5aezA	5b7yA
4u0oB	4v1kA	4wsfA	4xotA	4ytbA	4zldA	5afwA	5b89A
4u3yA	4v1sA	4wtpA	4xpxA	4ytdA	4zlfA	5afyH	5b8dA
4u5hA	4v29A	4wtxA	4xpzA	4ytkA	4zmkA	5afyL	5bjxA
4u5rA	4v33A	4wu0A	4xq7A	4ytwC	4zmyA	5agdB	5bk9B
4u7aA	4v3iA	4wuiA	4xqcA	4ytwD	4zo2B	5agiA	5bmnA
4u8fB	4v3lC	4wutA	4xrmA	4yucA	4zotA	5agrA	5bmtA
4u98A	4w5zA	4wuvA	4xslA	4yudA	4zoxA	5ah1A	5bobA
4u9hL	4w64A	4wwfA	4xtbA	4yvoA	4zoxB	5ahkA	5bovA
4u9hS	4w6yA	4wwhB	4xtlA	4ywaA	4zoyA	5aigA	5bowA
4u9oA	4w78C	4wy4A	4xtvB	4ywkA	4zpcA	5ailA	5bp3A
4u9uA	4w78H	4wy4B	4xuwA	4yx1A	4zqxA	5aimA	5bp9A
4ua6A	4w79A	4wy4C	4xxfA	4yycA	4zr8A	5ajgA	5bpkA
4ua8A	4w7lA	4wy4D	4xxlA	4yz0A	4zrxA	5ajoA	5br4A
4uabA	4w7wA	4wy9A	4xxxA	4yzgA	4zs9B	5akrA	5bs1A
4uasA	4w88A	4wydA	4xzfA	4yznA	4zurB	5al6A	5bsrA
4uavA	4w8bA	4wzxA	4y1bA	4yzrA	4zv0A	5an5J	5bt9A
4udgD	4w8hA	4wzxE	4y1wA	4yzzA	4zv0B	5anpA	5btwB
4udqA	4w8pA	4x00B	4y2fA	4z04A	4zv5A	5ao9A	5btyA

5bv8A	5cvwA	5e4gA	5favB	5gs7A	5htlA	5ix8A	5js4A
5bwiA	5cwbA	5e56A	5fbfA	5gsmB	5htxA	5ixbA	5jscA
5bxrB	5cwgA	5e5yA	5fc1A	5gt5A	5hubA	5ixgB	5jskB
5by5A	5cwhA	5e68B	5fc9B	5gtqA	5hw3A	5ixhA	5jugA
5by8A	5cwlA	5e75A	5fd9A	5gtuA	5hwaA	5iy2A	5juhA
5by8B	5cxxA	5e7hA	5febA	5gtuB	5hwkA	5iz3A	5jviE
5bykA	5cyvA	5e95B	5fewA	5gv0A	5hwnA	5izaA	5jxmA
5c04A	5czwA	5e9pA	5ffdA	5gv8A	5hwoA	5j1jA	5k08A
5c0pA	5d1mB	5ec6A	5fffA	5gvrA	5hzdA	5j1nA	5k26A
5c12A	5d2kA	5eckA	5ffxA	5gwnA	5i0yA	5j1sA	5k2iA
5c17A	5d4nC	5edfA	5fh7A	5gy7A	5i1uB	5j1sB	5k2lA
5c2uA	5d4vA	5eeqA	5fi3A	5gycA	5i29A	5j3tA	5k2xA
5c30A	5d5yB	5ehaA	5fisA	5gz3A	5i2hB	5j3tC	5k34A
5c33B	5d66A	5ehiA	5fjdA	5h0mA	5i32A	5j41A	5k3xA
5c3fB	5d6eA	5ej8A	5fjlA	5h0qA	5i34A	5j4fA	5k4bA
5c40A	5d78A	5el3A	5flwA	5h1nA	5i39A	5j4lA	5k6dA
5c4mA	5d7uA	5el9A	5flyB	5h28A	5i45A	5j4oA	5k7aA
5c5gA	5d7wA	5elbC	5fmuA	5h2dA	5i5bA	5j4uA	5k87A
5c5tA	5d84A	5em0A	5focA	5h3jA	5i5mB	5j6yA	5k8jA
5c5zA	5d8vA	5embA	5fpzA	5h3jB	5i5nA	5j90A	5k8jB
5c68A	5daeA	5emiA	5fqaA	5h3vA	5i7iA	5jajA	5k8sA
5c6sA	5dagA	5emxA	5fqeA	5h6tA	5i86A	5jazA	5k91A
5c79A	5dblA	5ep2A	5frdA	5h6xA	5i8fA	5jbnA	5karA
5c7hA	5dclA	5ep6A	5fs8A	5h7eA	5i90A	5jbxA	5kb6A
5c7qA	5dcuA	5ep6B	5fsvA	5h9iA	5i95A	5jc8A	5kdiB
5c8zA	5de3A	5epfA	5ftbA	5h9nA	5iaiA	5jcaL	5kdsA
5c98A	5dfyA	5epwA	5fu5A	5hb6A	5ib9A	5jcaS	5kf9A
5cd2A	5dgjA	5eq0A	5fuiA	5hb7A	5ibqA	5jdaA	5kfzA
5cdkA	5dhdA	5eq7A	5fukA	5hbpA	5icuA	5jddA	5khtA
5cdvA	5dicA	5er6A	5fv5A	5hbsA	5idbA	5jdkA	5ki9A
5cecA	5djhA	5etrB	5fvnD	5hc0A	5idhA	5je2B	5kkoA
5cecB	5dkaA	5eu0B	5fydA	5hdiA	5idqA	5jelA	5klaA
5cegB	5dkxA	5ew0A	5fypA	5hdmB	5idvA	5jelB	5kleA
5cegC	5dldA	5ewoA	5g1aA	5heeA	5ifzA	5jgkA	5knhI
5cfjA	5dleA	5ewuA	5g28A	5hgjB	5ig6A	5jgyA	5ko4A
5cg5A	5dloA	5ewyA	5g2uA	5hgwA	5igiA	5jh8A	5ko5A
5cgqA	5dltA	5ex2A	5g2vA	5hgzA	5ihfA	5jhxA	5ko9A
5cgqB	5dm2A	5exhC	5g38A	5hhaA	5ihwA	5ji7A	5kp7A
5civA	5dmaA	5ey0B	5g3yA	5hheD	5ii6A	5jicA	5kp7B
5ciyA	5dmdA	5eyfA	5g4iA	5hhjA	5ii8A	5jigA	5ktnA
5cklA	5dnlA	5eynA	5g51A	5hj1A	5ik4A	5jj2A	5kvbA
5cl8A	5dp2A	5ezuA	5g5cA	5hj9A	5imaA	5jkjA	5kvcB
5cm7A	5du9A	5f2kA	5ggbA	5hjfA	5in1A	5jlaA	5kvrA
5cmlA	5dufA	5f3mA	5ggnA	5hk3A	5inbB	5jlbA	5kvsB
5cofA	5dviA	5f47A	5gi7A	5hkoA	5io9A	5jmuA	5kwmA
5cowA	5dwaB	5f4cA	5gizA	5hl3A	5ipyA	5jntA	5kxhA
5coyA	5dxlA	5f5nA	5gjiA	5hmlA	5iqnG	5jo8A	5kxhB
5cozA	5dxxA	5f68A	5gkmB	5hnvA	5ir4A	5jodA	5ky0B
5cphA	5dzeA	5f6eA	5gm9A	5hoeA	5isvA	5jovA	5ky4B
5cr4A	5dzoA	5f6rA	5gmdA	5hpjA	5it6A	5jowA	5ky5B
5crwA	5dzsA	5f7vA	5gnfA	5hqhA	5itmA	5jp6A	5kycB
5ctaA	5e1nA	5f82A	5gngA	5hqjA	5itqA	5jphA	5kzzA
5ctdC	5e1wA	5fa8A	5gp7A	5hraA	5itwA	5jqfA	5l0nA
5ctmA	5e1yA	5faaA	5gqiA	5hsfA	5ivkA	5jqnA	5l0rB
5ctvA	5e37A	5fafA	5grqA	5hsgA	5iwhA	5jrtA	5l0vA
5cuoA	5e4bA	5fagA	5grqC	5ht2A	5iwuA	5jryA	5l0vB

5l20A	5m0nA	5n4bA	5o2xA	5qivA	5tw4B	5uyjA	5w8qA
5l20B	5m0wA	5n4bC	5o37A	5qoqA	5tw9A	5uzgB	5w98A
5l2lA	5m0yB	5n4kA	5o45A	5qr1A	5tz5A	5uzmA	5wa2A
5l37C	5m10A	5n6fA	5o58A	5qs9A	5tzmA	5uzxA	5wd9A
5l4lA	5m17A	5n7qA	5o5sA	5qu8A	5tzpA	5v01A	5wecA
5l6uA	5m1mA	5n81A	5o63A	5r0dB	5u00A	5v0mA	5wfbA
5l74A	5m1pA	5n86A	5o6hA	5r4oA	5u0iA	5v0zC	5wfyA
5l77A	5m29A	5nakA	5o6tA	5r4qA	5u1hA	5v1vB	5wgiA
5l87A	5m2oB	5nb4A	5o75A	5r4vA	5u23D	5v1vD	5wjpA
5l9zA	5m2pA	5ncbA	5o95A	5r7xB	5u2oA	5v1yB	5wk0A
5l9zB	5m33A	5ncjA	5o99B	5r8qA	5u3aA	5v2oA	5wkrA
5lalB	5m3qA	5ncwA	5o9mA	5rjjA	5u4hA	5v3nA	5wl1A
5lauA	5m4bA	5ncwB	5o9qA	5rkzA	5u4nA	5v3nB	5wljB
5lb7A	5m5zA	5nfmA	5oakA	5rw1B	5u4qA	5v44A	5wm2A
5lb7B	5m72A	5nfqA	5oazA	5suiA	5u4sA	5v5hA	5wmkA
5lbdA	5m72B	5ng7A	5obpA	5sv2A	5u5oA	5v6fA	5wn9A
5ldgA	5m7yA	5nggA	5obtE	5sv5A	5u5tC	5v6jA	5wouA
5leoA	5m97B	5ngnA	5obyA	5svyA	5u69A	5v89A	5wp4A
5lfzA	5maoA	5nhuJ	5od4A	5swcE	5u7aA	5v8sA	5wqjA
5lhmA	5mawD	5ni9A	5odkA	5sy4B	5u81A	5vbdA	5wriA
5lhwA	5mawE	5ni9B	5oduE	5szcA	5u8uA	5vcmA	5wsfA
5lhxA	5mbxA	5nioA	5oe3A	5t1iA	5uamA	5vczA	5wucA
5li7A	5mc7A	5nj9A	5of1A	5t39A	5ubaA	5veiA	5wwdA
5ljlA	5mduA	5nj9B	5ofkA	5t3bA	5ucsB	5vf5A	5wxhA
5ljmA	5mfaA	5njiA	5ohqA	5t46B	5udiA	5vfaA	5x2eA
5ljpA	5mfoA	5njoA	5oj5A	5t5lA	5ue1A	5vfbA	5x40A
5ljxA	5mgwA	5nldB	5oj7A	5t7aA	5uebA	5vg0A	5x4bA
5lkbA	5mh6A	5nmnA	5ojlA	5t7dB	5uejA	5vg3B	5x4rA
5lndA	5mjrA	5nmxA	5ok6A	5t8cA	5ufhA	5vgbA	5x57A
5lnnA	5mk9A	5nnaA	5okaA	5t9cE	5ufnA	5vgbB	5x5jA
5lomA	5ml3B	5noaA	5ol4A	5ta0A	5ufyA	5vglA	5x5mA
5lp9A	5mozA	5nqoA	5ol4B	5tabA	5ug9A	5vhgA	5x5vA
5lpaA	5mprA	5nr4B	5ol4C	5tcbA	5ugrA	5vi6A	5x7lA
5lq5A	5mpwA	5nrhB	5ol9A	5tdaA	5ujcA	5vivA	5x89A
5lq6A	5mr1A	5nrmA	5ollA	5tdrA	5ul6A	5vjtA	5x9iA
5ls4A	5msaA	5nrmB	5olrC	5tfqA	5ulbA	5vn4B	5x9lA
5ls7B	5msoA	5nsaA	5omtA	5tg0A	5um2A	5vnyA	5xa5A
5ls7D	5mszA	5nt7A	5on8B	5thkA	5umfA	5vogA	5xa5B
5lsvA	5mteA	5nt7B	5onkA	5tifA	5umhA	5vpsA	5xavA
5lt5A	5mu9A	5ntbA	5opfA	5tjzA	5umpA	5vrkA	5xb0A
5ltlA	5muaB	5nulA	5opzA	5tk2C	5umrA	5vscA	5xbcA
5lu5D	5mujA	5nuvA	5oq3A	5tkwA	5umsA	5vugA	5xbiA
5lunA	5mulA	5nvjA	5otnA	5tkzA	5uouA	5vx1B	5xbuA
5lusA	5mwaA	5nw3A	5ouoA	5tleA	5uq6A	5vxvA	5xc5A
5lvoA	5mwzA	5nwpA	5ovkA	5tnvA	5uqsC	5vyqA	5xcoB
5lw3A	5mx9A	5nx7A	5ovoA	5tnwB	5uqzA	5w0gA	5xcqB
5lwxA	5my5A	5nykA	5ovvA	5toqA	5ut3A	5w0hA	5xdcA
5lx6A	5my7A	5nzgA	5oxzA	5tpiA	5uuiA	5w2fA	5xdhC
5lxeA	5mzwA	5nzoB	5oxzB	5tqiA	5uukA	5w2iA	5xecA
5lxxA	5mzwD	5o0dA	5oycA	5tqjA	5uukB	5w3rA	5xevA
5lxzB	5n0oA	5o0sA	5p9jA	5trqA	5uuoB	5w4aC	5xj5A
5ly3A	5n17A	5o0uA	5p9vA	5tsqA	5uwaB	5w7wT	5xk6A
5ly8A	5n1pA	5o15A	5q22A	5tt5A	5uwzA	5w83A	5xkaA
5lypA	5n3jA	5o1lA	5qhhA	5tv2A	5ux1A	5w83B	5xkrA
5lzkA	5n41A	5o29A	5qi0A	5tvoA	5uxmA	5w8jA	5xkxA
5lznA	5n48B	5o2dA	5qinA	5tvoB	5uxsA	5w8oB	5xluA

5xluB	5yseB	6a27A	6blkD	6d0hB	6efcA	6fc0B	6g44A
5xm5B	5ysiA	6a56B	6bm5A	6d4kA	6efnA	6fc1B	6g47A
5xn9B	5ysqA	6a58A	6bnzA	6d4rA	6egeA	6fdgA	6g49A
5xneB	5yt6F	6a5dB	6bo0A	6d9nA	6eh4D	6fdkA	6g4jA
5xtuA	5yufA	6a71A	6bqaA	6d9yA	6eh4E	6fexA	6g4jB
5xveA	5yugA	6a7tB	6bscA	6dcdA	6ehbA	6ff1A	6g5pA
5xvjA	5yuqA	6a80B	6bscB	6dceA	6ehiA	6ff2A	6g62A
5xvtA	5yvkA	6a9sA	6bsuA	6dcjA	6eimA	6ffaA	6g6kA
5xw2A	5yvnA	6ac0A	6bt1A	6dcmA	6eioA	6fg8A	6g6kD
5xwxA	5yvxA	6ac5A	6bumD	6ddmC	6ekgY	6fg8B	6g7nA
5xz4A	5ywrB	6ae9A	6bw9A	6dfpA	6eklB	6fgcA	6g85A
5xz7A	5yxgA	6ag8C	6bw9B	6dg4A	6ekzA	6fggA	6g8uA
5y0mA	5yxmA	6aibA	6bwlA	6dgaA	6elcA	6fi2A	6g8yA
5y1fA	5yzpA	6ajpA	6bxdA	6dggA	6elmA	6fieB	6g96A
5y33A	5z0dA	6akkA	6bxgA	6dgmB	6elvA	6fihA	6gajA
5y46A	5z0dB	6am3X	6bxrA	6dhtA	6eniA	6fiyB	6gbiA
5y4mA	5z0uA	6amgB	6c10A	6dkqA	6ensA	6fj7A	6gcfA
5y4tA	5z37A	6anzA	6c1xA	6dnmA	6eozA	6fjnA	6gcvA
5y4zA	5z3eA	6ao9A	6c1zA	6dnoB	6eqeA	6fjvA	6gd3A
5y5qA	5z42A	6apeA	6c29B	6dopA	6eqsA	6fl1A	6gdjA
5y6yB	5z48A	6aqsA	6c2cB	6dqhA	6er1A	6flfA	6gdxB
5y90A	5z51A	6ar0A	6c30B	6dqpB	6er4B	6flkA	6gehA
5y9xA	5z6bA	6arhA	6c3cA	6ds9A	6es9A	6fm5A	6geuA
5y9zA	5z6dA	6at4B	6c3mA	6dt3A	6et0A	6fm7A	6gg1A
5ya6A	5z99A	6atrA	6c4qA	6dtsA	6et0B	6fmbA	6gg7B
5yalA	5z9yA	6atwA	6c52D	6dtvA	6etlA	6fmcA	6gg7C
5yayA	5za3A	6avxA	6c5bA	6dubA	6eu8A	6fmeB	6ggpA
5yayB	5zb0A	6b1kA	6c74A	6dvrA	6euwA	6fnuA	6ghtA
5ybyA	5zbfA	6b1zA	6c8cB	6dyfA	6evgA	6fohA	6gi2A
5yc6U	5zbyA	6b26A	6c9xA	6e0kA	6evnA	6fopA	6gi4B
5ycaC	5zbzA	6b2vA	6cafA	6e0oA	6evuA	6foqA	6gitA
5yceA	5zcyA	6b5kA	6caxA	6e1fB	6ewhA	6fpqA	6gkeA
5yddA	5zdmA	6b6uA	6cb7A	6e1xA	6ewlA	6fq1A	6gkxA
5ydeA	5ze8A	6b7pB	6cbnA	6e1zA	6ewmA	6freA	6gmcA
5ydnA	5zhoA	6b8fA	6cbrA	6e28C	6exmA	6fsgA	6gmfA
5yedA	5zhzA	6b9hA	6cbuA	6e3aA	6exxA	6fsnA	6gmpA
5ygbA	5ziqA	6b9hB	6cd7A	6e3iB	6exzA	6ft2A	6gn5A
5yh4A	5zkeB	6b9xA	6cd9A	6e4dA	6ey1A	6ftfB	6gnaA
5yhrA	5zm0A	6b9xB	6cdxB	6e4lA	6eygA	6fthA	6gp3A
5yiuA	5zmuA	6b9xC	6chxA	6e55D	6eziA	6ftoA	6gpkA
5yj6A	5zo3A	6b9xD	6cj7A	6e5fA	6f0wS	6ftoC	6gpzB
5ykjA	5zqaA	6b9xE	6ckaB	6e5xA	6f43A	6fu9A	6gqcA
5ykrA	5zrcA	6ba9A	6ckmA	6e5yA	6f4jA	6fu9B	6gqdA
5ykuA	5zryA	6bcbA	6cngD	6e60A	6f4jC	6fucA	6gqzA
5ykzA	5zt3A	6bcdA	6cnwA	6e68A	6f5cA	6fviA	6greB
5ylgA	5zu6A	6bcdB	6cojA	6e6oA	6f5zA	6fw0A	6grlA
5ynxB	5zw7A	6bd0A	6cojB	6e6qB	6f5zC	6fxaA	6gszA
5yobA	5zwuA	6bdnA	6cpbB	6e6uB	6f6mA	6fxdA	6gugA
5yofA	5zx8A	6bevA	6cr0A	6e7eA	6f8aA	6fyjA	6gv5A
5yqaA	5zx9A	6bg8B	6ctzA	6e85A	6f8bA	6fyrA	6gv8A
5yqjA	5zzaP	6bgdA	6cumA	6e94A	6f8nA	6fz6A	6gvdA
5yqwA	6a02A	6bgyA	6cw0A	6ectA	6f8pA	6g00A	6gvkB
5yrhA	6a0aA	6bhdA	6cwmA	6edmA	6f9oA	6g1cV	6gx2A
5yrvA	6a0cA	6bioA	6cz4A	6edvA	6f9qA	6g1iA	6gy5A
5yrvB	6a0jA	6bjbA	6d0aA	6ef6A	6fbqA	6g1pA	6gz0A
5yrvC	6a1iA	6bk0A	6d0hA	6ef7A	6fc0A	6g25A	6gz8A

6gzuA	6hurA	6j4pB	6kc5B	6lqfA	6ne2A	6ozdA	6q5aA
6gzwA	6hx0A	6j6pA	6ketA	6lqnA	6nfrA	6ozeA	6q5oA
6h0cA	6hxmA	6j6vA	6kfaA	6luhB	6nhxA	6p0cA	6q61A
6h0hB	6hxpA	6j93A	6kfnA	6lutB	6nibA	6p28A	6q62A
6h0mA	6hy3A	6j98A	6kfsA	6lxaA	6nioA	6p29B	6q6rG
6h10A	6hyfA	6jalA	6kgcA	6m0eA	6nj1A	6p2lA	6q6tA
6h17A	6hyoA	6jccA	6kgcB	6m1vA	6njkA	6p2nA	6q78A
6h1qA	6hzgA	6jchA	6khlA	6m3dC	6nk0B	6p3nA	6q7dA
6h20A	6hzrA	6jd9A	6kiaB	6m4qA	6nkhD	6p44B	6q7iB
6h24A	6i03A	6jebA	6kiiA	6m64A	6nlqC	6p58B	6q7rA
6h2rC	6i05A	6jh7A	6kilA	6m64F	6nnrB	6p5hB	6q8eA
6h2uA	6i0iA	6jiwA	6kisA	6m76A	6np3A	6p7zA	6q9lA
6h2uB	6i18A	6jjtD	6kjkA	6m8nA	6nq4B	6p80A	6qazA
6h40A	6i1aB	6jk2A	6kjwA	6m9mA	6nq6A	6p8jB	6qdiA
6h4eA	6i1mA	6jk4A	6klzA	6m9zD	6nq6D	6pbmA	6qe0A
6h4lA	6i3bA	6jkrF	6kmjA	6maaA	6nrhA	6pcdD	6qhgA
6h5wA	6i3qB	6jkzA	6kmoB	6mb8A	6nsvA	6pckA	6qhjA
6h8gA	6i4eA	6jl3A	6ko8A	6mbbB	6nvxA	6pczA	6qjaA
6h8nA	6i4eG	6jleA	6kp5B	6mbfA	6nvxB	6pfxB	6qjlA
6h8oA	6i5oD	6jleE	6kqsA	6mdhA	6nx0A	6phiA	6qlaB
6h96A	6i5rA	6jm5B	6kr5B	6mdwA	6nx5C	6pjvA	6qo9B
6h99A	6i65A	6jmbA	6krwA	6mfuA	6nyoA	6pkfA	6qp1A
6h9uB	6i6mA	6jnjA	6ksrA	6mgcA	6nytA	6pkhA	6qpkA
6ha4A	6i8yA	6jnyB	6kthA	6mhaA	6nzsA	6pljA	6qprA
6harE	6i9aA	6jptA	6ku0B	6micA	6o15B	6pnvA	6qpsA
6havA	6i9oC	6jq8A	6kv9A	6mihA	6o19A	6pqkA	6qspA
6hazA	6i9wA	6jqbA	6kwzA	6mj7A	6o2vA	6pqkD	6qspB
6hbbA	6ibeA	6jqkC	6kxtA	6mm2A	6o3pA	6pt4B	6qtsA
6hc1B	6icgA	6jsaA	6kzjA	6mroA	6o40A	6pt8B	6qvfD
6hcwA	6iewA	6jtbA	6kzjC	6mrqI	6o40B	6puqA	6qvsA
6herA	6if3A	6ju1A	6l0oA	6mrrA	6o4mA	6puvA	6qw0B
6hfmA	6ifbA	6ju8B	6l0qD	6mrsA	6o5iA	6pvjA	6qxrA
6hfqA	6ifpA	6jueL	6l0vE	6mu0A	6o5kA	6pwsA	6qxuA
6hg7A	6iggA	6jufB	6l1pA	6mw4A	6o6jA	6px0A	6qz3A
6hgmB	6ih0A	6jv0A	6l27A	6mwsA	6o8lA	6pxcA	6r09A
6hh2A	6ihrA	6jv7A	6l4vA	6mx3A	6o9sA	6pymA	6r0rA
6hheA	6iipA	6jvvA	6l5hB	6mydA	6oalA	6pz7A	6r1dB
6hhmA	6iiyA	6jwfA	6l69B	6myiD	6od3F	6pzdA	6r1gB
6hhnA	6ij1A	6jwjA	6l7qA	6n0dB	6ofqA	6pzlA	6r1hA
6hihA	6ijeB	6jwjC	6l8gA	6n0kA	6ohkA	6q00A	6r2iB
6hipB	6imeB	6jwmA	6l8gB	6n0xC	6oj7A	6q00B	6r2wL
6hiuB	6inxA	6jyzA	6l8sA	6n1bA	6ojfB	6q10A	6r2wT
6hk9B	6iqcA	6jz2A	6l8xA	6n36A	6ojlA	6q1hG	6r33A
6hl1A	6iqxA	6k0pA	6lacA	6n3dA	6ojmA	6q1mA	6r3mA
6hlyA	6itaA	6k1tA	6lanA	6n4lA	6om5A	6q2pB	6r3wA
6hn1A	6itgA	6k1wA	6ld1A	6n59A	6on6A	6q3pA	6r4zA
6hniA	6iuxA	6k2fB	6ldlA	6n6jA	6oodA	6q3pB	6r5jC
6hpaA	6iwvB	6k39A	6lebB	6n7aA	6oriA	6q3pC	6r5tA
6hpfA	6ix1B	6k4xA	6lf2B	6n87A	6os6A	6q41A	6r62A
6hphA	6ix2B	6k5gD	6lftA	6n9bA	6os7B	6q41B	6r7vA
6hqcA	6iy4I	6k7cA	6lg2B	6n9hA	6osxA	6q43A	6rb4A
6hs0A	6j0eB	6k82B	6lgiB	6n9iA	6ovmB	6q43B	6rb7F
6hsaA	6j33B	6k93A	6ljeB	6n9mA	6ovmR	6q43C	6rccX
6hsdA	6j4dA	6k9jA	6lk1A	6nauA	6owdA	6q4gA	6rg2B
6hsjA	6j4kB	6kbxB	6lkkA	6nd7A	6oxjB	6q4rA	6rhfA
6htoA	6j4pA	6kc4E	6ll8A	6ndtB	6oz7E	6q4zB	6ri6A

6rimH
6rivA
6rjiA
6rk0A
6rlxC
6rlxD
6rnvA
6rnzB
6ro6F
6rppA
6rraA
6rrvA
6rs4B
6ru1A
6rvqA
6rvuA
6rw0A
6rw7A
6rwtA
6rxaA
6rxkA
6ry0A
6ry3A
6rygA
6ryzB
6rz0A
6s07A
6s0pA
6s1hA
6s2mA
6s2rA
6s33A
6s5kA
6s5wB
6s6cA
6s6fB
6s8kB
6s95B
6s98B
6s9kB
6sa5A
6saoA
6sbaA
6sbaB
6sbfA
6sbqA
6scbB
6scfC
6scqA
6sd8X
6se1A
6shuA
6sidA
6sigD
6sj3A
6sj8A
6sjaB
6sldA
6sllB
6smtC
6sooA
6sp9A
6spoA
6sqpB
6sqxB
6sreA
6srhA
6srnA
6srtA
6ssdA
6sshA
6st4A
6stlB
6su3A
6su5A
6sunA
6svlF
6swiA
6swtB
6sxtA
6sygA
6syiA
6syjB
6syvA
6t02B
6t0kA
6t0yA
6t1uB
6t2tA
6t3xA
6t3zA
6t4xA
6t5jB
6t5kC
6t6eA
6t6hA
6t6kA
6t7oA
6t84A
6t9qA
6tabA
6tbiF
6tcbB
6tccA
6tciA
6tcvB
6teqB
6tfrB
6tfxB
6tg6A
6tgjA
6tgkC
6tgsA
6tguA
6thoA
6tj2B
6tj4A
6tjhA
6tjrA
6tklI
6tl7B
6tm3A
6tm9A
6tq5D
6tqtA
6tr4A
6trjA
6trkB
6ts4A
6tt2B
6ttnA
6tv2D
6tveP
6twtB
6tyjA
6tyuA
6tyyA
6tzlD
6tznA
6tzxA
6u10A
6u2sA
6u2uB
6u43A
6u4zA
6u54B
6u66A
6u97A
6uaqA
6uawA
6uaxA
6ub1D
6ub6A
6ubdA
6ublB
6uboA
6udvA
6uf3A
6ufaA
6ufeA
6ufvA
6uidA
6ujkB
6ukfX
6ullA
6uloA
6umuA
6uofA
6uq8A
6uqvA
6uscB
6ut9A
6utcA
6uwaA
6uwpB
6uwwA
6uxeA
6uxeB
6uxeD
6uy5B
6v04A
6v1cA
6v2sA
6v42B
6v4mA
6v4wB
6v67B
6v71A
6v7gA
6vagB
6veaA
6vg5B
6vgwA
6vh6A
6vhuA
6vigD
6vjjB
6vjuA
6vk6A
6vk6B
6vk6C
6vo5B
6vo5D
6vo6C
6vtbA
6vtmE
6vvnB
6vydA
6vz9A
6vzaA
6vzxB
6w0vA
6w0vB
6w1gA
6w3dA
6w3wA
6w46C
6w47B
6w4lA
6w70A
6w90A
6w9oA
6wayA
6wcdA
6wcvB
6wesA
6wfiA
6wfnA
6wgmA
6widA
6wm6A
6wmdA
6wmdB
6wmm
A
6wn5A
6wn9B
6wngB
6wqyA
6wrtA
6wt8A
6wupA
6wxoA
6x1gA
6x1nA
6x1xA
6x42X
6x6zA
6x7nA
6x84A
6x8oA
6x94A
6xb6A
6xd8B
6xfjB
6xfuB
6xhhB
6xicA
6xicB
6xigA
6xipB
6xipC
6xjeD
6xmiC
6xmyA
6xokA
6xpjC
6xrkB
6xsoB
6xvmB
6xweA
6xxjA
6xy1C
6xyaB
6xybA
6xzuB
6y1wB
6y1yB
6y43A
6y4eA
6y56A
6y5uA
6y74A
6y9jA
6y9qB
6ya6A
6ya6B
6ycbA
6ydrA
6yfiA
6yiiA
6yipA
6yk4A
6yp6A
6ypeA
6yskA
6ywnA
6z1vA
6z30A
6z4aA
6z4wA
6z96A
6z9kA
6zcoA
6zeaA
6zegB
6zegC
6zjeA
6zjsA
6zlkC
6znvA
6zpeA
6zpkA
6zrnC
6ztxA
6zxtA
6zzoC
7a19B
7a3hA
7a3mA
7a5mA
7a77A
7abbA
7adrA
7adrE
7adrF
7agmA
7akcA
7akyA
7aniB
7ao3B
7bqgA
7bqiA
7bqpA
7bu2B
7bvtA
7bwhA
7bysB
7bzkA
7bzkB
7c0dK
7c1iA
7c1uA
7c31A
7c38B
7c5zA
7c7dB
7c82A
7c8fA
7c8sA
7cbdA
7cboA
7cflC
7cfwA
7ckjA
7cn7A
7cn7C
7cncB
7d3dB
7fd1A
7jidA
7jjkA
7jp2B
7k3tA
7kb2A
7kffA
7kpoA
7kr0A
7odcA
8abpA
