# Supplementary material for: Automated Protein Secondary Structure Assignment from Cα Positions Using Neural Networks
Source: Biomolecules. 2022 Jun 17;12(6):841. doi: 10.3390/biom12060841 (PMC9220970; doi:10.3390/biom12060841)
Supplement: Supplementary file 1 [file biomolecules-12-00841-s001.zip › SUP3-hbonds_for_heca.pdf]

Hydrogen bonds potential

A hydrogen bond, which is a non-local (in the sense of sequence separation) potential is necessary to render proper packing of β strands in a sheet. We present a hydrogen bond potential devised to assess the geometry of β strands in proteins based only on Cα positions. The interaction involves two centers i and j, each of them with three subsequent α carbons: $i-1, i, i+1$ and $j-1, j, j+1$, respectively. Spatial arrangements of these atoms may be seen as two triangles, referred further as triangle i and triangle j. The general idea of the potential is to *(a)* properly arrange the two triangles i and j in space, and *(b)* to ensure each triangle interacts with at most two other triangles and only one on each side.

To detect an H-bond between i-th and j-th atoms, we use local coordinates systems (LCS) built on the central atoms of each triangle, i.e. three consecutive Cα atoms centred on i-th and j-th atoms, respectively. Formulas to calculate an LCS are given below:

$$\begin{aligned}\vec{v}_x &= \vec{v}_{1,2} + \vec{v}_{2,3}\\ \vec{v}_y &= \vec{v}_z \times \vec{v}_x\\ \vec{v}_z &= \vec{v}_{1,2} - \vec{v}_{2,3}\end{aligned}$$

where $\vec{v}_{l,m} = |\vec{C\alpha}_m - \vec{C\alpha}_l|$ is a versor pointing from l-th to m-th Cα atom. In the next step, we calculate local coordinates of Cα_i in the LCS of triangle-j and *vice versa*.

Statistics of these local x, y, z coordinates computed from a representative subset of PDB structures (PISCES). Analysis show that it is reasonable to change the cartesian coordinates y and z to radial r and angle. So instead of x, y, z we will use x, r and α (i.e. the angle). After analysis of the statistics we decided to approximate each variable by a normal distribution. Parameters of these distributions were estimated with truncated robust estimator: 10% of extreme values were removed from estimating μ and σ parameters of each Gaussian function. All these analyses and estimations were computed with BioShell package.

According to our definition, a CG hydrogen bond can be detected if all the six variables: x_i, r_i, α_i, x_j, r_j and α_j (three local coordinates of i-th Cα defined in the LCS of j-th triangle and three local coordinates of Cα_j defined in the LCS of triangle i, respectively) are in the allowed range established as described above. The final energy value is defined as:

$$\begin{aligned}E(x_i, r_i, \alpha_i, x_j, r_j, \alpha_j) = [&f(x_i; \mu_x, \sigma_x) * f(r_i; \mu_r, \sigma_r) * f(\alpha_i; \mu_\alpha, \sigma_\alpha)\\ &*f(x_j; \mu_x, \sigma_x) * f(r_j; \mu_r, \sigma_r) * f(\alpha_j; \mu_\alpha, \sigma_\alpha)]^{1/6}\end{aligned} \tag{S1}$$

The parameters for the equation:

	Antiparallel		Parallel	
	μ	σ	μ	σ
x, $\alpha > 5$	-0,512	0,300	0,078	0,241
r, $\alpha > 5$	4,748	0,174	4,796	0,146
α, $\alpha > 5$	6,524	0,216	6,429	0,160
x, $\alpha < 5$	-0,715	0,304	-0,075	0,300
r, $\alpha < 5$	4,812	0,128	4,792	0,164
α, $\alpha < 5$	3,375	0,243	3,272	0,175

The above equation is just a geometric average of fitness of each internal coordinate to the respective distribution observed in experimental structures. The sigmoid $f()$ function is applied to make the energy function derivable. The energy value is symmetric: when atom Cα_i is H-bonded to Cα_j, at the same time Cα_j interacts with Cα_j. The detailed algorithm to calculate a HB between i-th and j-th Cαatoms is as follows:

- calculation of distances in every pairs (i-1, j-1) (i, j) and (i+1, j+1) for parallel and (i-1, j+1)(i, j) and (i+1, j-1) for antiparallel - this distances allows to distinguish the type of a sheet; all these distances must be in the range of $4.0 \leq d \leq 6.2$
- calculation of LCS at Cα_i and Cα_j according to formulas above
- evaluation the energy value according to the formula (S1) with parameters from the table above
